# Zonally patterned demineralized bone matrix–meniscus ECM composite scaffold directing region-specific fibrochondrogenic and angiogenic responses

**DOI:** 10.1093/rb/rbag081

**Published:** 2026-04-26

**Authors:** Hee-Woong Yun, Chae-Won Yun, Mi Jeong Kim, Yeeun Kim, Gaeun Shim, Sujin Noh, Ho Jin Lee, Sumin Lim, Jun Young Chung, Jae-Young Park, Do Young Park

**Affiliations:** Department of Orthopedic Surgery, School of Medicine, Ajou University, Suwon 16499, Republic of Korea; Cell Therapy Center, Ajou University Medical Center, Suwon 16499, Republic of Korea; Cell Therapy Center, Ajou University Medical Center, Suwon 16499, Republic of Korea; Ajou University, Leading Convergence of Healthcare and Medicine, Institute of Science & Technology (ALCHeMIST), Suwon 16499, Republic of Korea; Cell Therapy Center, Ajou University Medical Center, Suwon 16499, Republic of Korea; Ajou University, Leading Convergence of Healthcare and Medicine, Institute of Science & Technology (ALCHeMIST), Suwon 16499, Republic of Korea; Cell Therapy Center, Ajou University Medical Center, Suwon 16499, Republic of Korea; Ajou University, Leading Convergence of Healthcare and Medicine, Institute of Science & Technology (ALCHeMIST), Suwon 16499, Republic of Korea; Cell Therapy Center, Ajou University Medical Center, Suwon 16499, Republic of Korea; Ajou University, Leading Convergence of Healthcare and Medicine, Institute of Science & Technology (ALCHeMIST), Suwon 16499, Republic of Korea; Cell Therapy Center, Ajou University Medical Center, Suwon 16499, Republic of Korea; Department of Biomedical Sciences, Graduate School, Ajou University, Suwon 16499, Republic of Korea; Cell Therapy Center, Ajou University Medical Center, Suwon 16499, Republic of Korea; Department of Orthopedic Surgery, School of Medicine, Ajou University, Suwon 16499, Republic of Korea; Department of Orthopedic Surgery, School of Medicine, Ajou University, Suwon 16499, Republic of Korea; Department of Orthopaedic Surgery, CHA University, CHA Bundang Medical Center, Seongnam 13496, Republic of Korea; Department of Orthopedic Surgery, School of Medicine, Ajou University, Suwon 16499, Republic of Korea; Cell Therapy Center, Ajou University Medical Center, Suwon 16499, Republic of Korea; Ajou University, Leading Convergence of Healthcare and Medicine, Institute of Science & Technology (ALCHeMIST), Suwon 16499, Republic of Korea

**Keywords:** meniscus tissue engineering, decellularized extracellular matrix, composite scaffold, fibrochondrogenic differentiation, angiogenesis

## Abstract

The human meniscus is a zonal fibrocartilage characterized by inner–outer gradients in matrix composition and outer vascularity, essential for mechanical function and tissue integration. However, these hierarchical features are rarely reproduced in current meniscal substitutes, limiting durability and biological integration after repair. Here, we developed a triphasic scaffold composed of a demineralized bone matrix (DBM) framework integrated with regional decellularized meniscus extracellular matrix (DMECM) using riboflavin-mediated photo-crosslinking. This strategy enabled spatial localization of zonal DMECM compartments within a robust scaffold. The zonally patterned DBM + DMECM scaffold preserved interconnected porosity and mechanical stability, supported mesenchymal stem cell (MSC) adhesion and guided region-dependent fibrochondrogenic differentiation *in vitro*. Subcutaneous implantation of MSC-seeded scaffolds resulted in organized ECM remodeling and a graded angiogenic response across the inner, middle and outer regions. *In vitro* endothelial assays confirmed that these vascular patterns arise from intrinsic zone-specific DMECM cues, recapitulating the native avascular-to-vascular hierarchy. Together, these findings demonstrate that the triphasic DBM + DMECM scaffold restores biochemical and angiogenic gradients of the native meniscus, enabling coordinated fibrochondrogenic and vascular responses. The use of clinically established materials supports the translational potential of this platform for post-meniscectomy defect reconstruction and fibrocartilaginous interface engineering.

## Graphical abstract

**Graphical Abstract:**
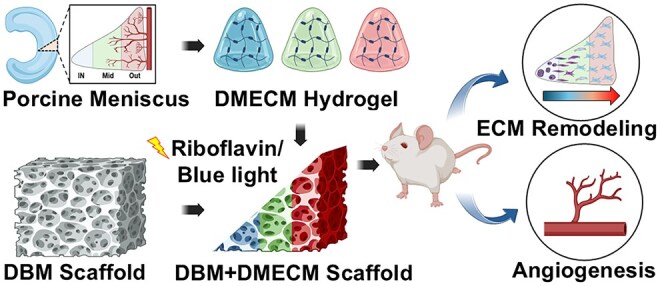

## Introduction

Arthroscopic partial meniscectomy (APM) is performed more than 500 000 times annually in the United States alone, providing short-term symptom relief but accelerating osteoarthritis progression by removing load-bearing fibrocartilage without restoring function [1]. There is a clinical need to regenerate the tissue void created during APM to restore the native load-bearing function of the meniscus.

Anatomically, the fibrocartilaginous meniscus is organized into inner, middle and outer zones that differ in extracellular matrix (ECM) composition, with the inner region exhibiting more cartilage-like ECM and the outer region displaying fibrous tissue [2, 3]. The inner zone is rich in proteoglycans and type II collagen and supports chondrogenic phenotypes but resists angiogenic ingrowth [2, 4]. In contrast, the outer zone contains dense type I collagen and supports fibrovascular integration, while the middle zone provides a biochemical and mechanical gradient connecting these extremes [2, 3]. This spatial heterogeneity is essential for function but is not reproduced in current meniscal implants, including collagen scaffolds and synthetic polymer constructs, which lack coordinated biochemical and vascular cues needed for long-term integration [5].

Decellularized meniscus ECM (DMECM) preserves native matrix complexity and zone-specific signals, offering a promising route to recapitulate regional cues [6–8]. However, DMECM hydrogels alone lack mechanical integrity [9], while synthetic scaffolds lack biochemical fidelity [5]. Thus, a strategy integrating structural robustness with zonal biochemical specificity is required to mimic the native meniscal microenvironment.

In this study, we developed a triphasic scaffold composed of a demineralized bone matrix (DBM) framework coated with zone-specific DMECM hydrogels using riboflavin-mediated photo-crosslinking. DBM provides a mechanically stable, porous collagen network biocompatible with cell infiltration and suitable for clinical translation [10, 11], while the DMECM coatings supply region-appropriate biochemical cues [6–8]. By spatially patterning DMECM derived from the inner, middle and outer meniscal zones, this approach aims to recreate native gradients in fibrochondrogenic potential and angiogenic regulation.

We hypothesized that a zonally patterned DBM + DMECM composite scaffold would (i) reproduce native biochemical heterogeneity, (ii) guide MSCs toward region-specific fibrochondrogenic phenotypes and (iii) modulate angiogenesis in a gradient consistent with the native meniscus. To test this, we characterized scaffold physicochemical properties, assessed MSC and endothelial responses *in vitro*, and evaluated ECM remodeling and neovascularization *in vivo*.

## Materials and methods

### Reagents and biological materials

All chemicals were purchased from Sigma-Aldrich (St. Louis, MO, USA) and cell culture reagents from Life Technologies (Carlsbad, CA, USA). Porcine meniscal tissues were collected within 6 h after sacrifice from 6-month-old Yorkshire pigs (Farmsco, Seongnam, Korea). Human DBM and synovial mesenchymal stem cells (MSCs) were obtained with approval from the Institutional Review Board of Ajou University School of Medicine (IRB No. AJOUIRB-SM-2025-051). All animal and human procedures were approved by the Institutional Animal Care and Use Committee of Ajou University (IACUC No. 2023-0086).

### Preparation of the DBM + DMECM scaffold

#### Preparation of DBM scaffold

DBM scaffolds were prepared from freeze-dried human cancellous bone cubes (Resource Tissue Bank, Seoul, Korea) as previously described [12]. Samples were sequentially demineralized in 5% nitric acid (120 rpm, 48 h, 25°C), decellularized in 15% Triton X-100 (72 h), washed several times with distilled water (DW), delipidized in ethanol (24 h) and freeze-dried, then stored at −80°C until use.

#### Preparation of zone-specific DMECM hydrogel

Porcine menisci were harvested within 6 h after sacrifice and divided into inner, middle and outer regions based on anatomical landmarks [6]. The tissues were lyophilized (72 h), pulverized using a cryogenic grinder (6870; SPEX, USA) and decellularized by sequential treatment with hypotonic buffer, 0.5% SDS and DNase, followed by repeated DW washes. Decellularized DMECM powders were lyophilized, filtered (100 μm), sterilized with ethylene oxide gas and stored at −80°C.

DMECM powders from each region—whole (DMECM-W), inner (DMECM-I), middle (DMECM-M) and outer (DMECM-O)—were digested at 2% (w/v) in 0.5 M acetic acid containing 0.1% pepsin (200 rpm, 37°C, 24 h). The digests were neutralized to pH 7.4 to obtain DMECM solutions, then mixed with 0.05% riboflavin (RF) and photo-crosslinked under blue light (405 nm, 3.5 mW/cm^2^, 2 min) to form stable hydrogels.

#### Preparation of the DBM + DMECM composite scaffold

DMECM hydrogels were loaded onto DBM scaffolds through five sequential loading–crosslinking–lyophilization cycles. In each cycle, 1 μL of 2% pre-DMECM hydrogel per mm^3^ of scaffold was applied, infiltrated for ∼10 s, photo-crosslinked (2 min) and lyophilized.

For zone-specific constructs, triangular prism-shaped DBM scaffolds were fabricated by diagonally cutting cuboidal blocks (12 mm × 10 mm × 5 mm). The sloped surface was divided into three 4 mm-wide regions corresponding to the inner, middle and outer zones. Zone-specific pre-DMECM hydrogels (DMECM-I, DMECM-M and DMECM-O) were sequentially applied and photo-crosslinked under identical conditions.

### Characterization of the DBM + DMECM scaffold

#### Scanning electron microscope

Microstructures of native bone, DBM and DBM + DMECM scaffolds (0–6 coating cycles) were examined with a field-emission scanning electron microscope (SEM; JSM-6700F; JEOL, Tokyo, Japan). Cross-sections were sputter-coated with gold and imaged at ×45 magnification.

#### Energy dispersive X-ray spectroscopy

Elemental compositions of native bone and DBM were analyzed using an energy dispersive X-ray spectroscopy (EDX) detector coupled to the SEM system (*n* = 5).

#### Porosity and pore size

Porosity and pore size of DBM + DMECM scaffolds with varying coating cycles were quantified from SEM images using ImageJ software (NIH, Bethesda, MD, USA) (*n* = 6).

#### Histological evaluation

Samples were fixed in 10% formalin (24 h, 4°C), dehydrated and embedded in paraffin. Sections (4 μm) were stained with hematoxylin and eosin (H&E) to assess decellularization and ECM preservation. For bone-derived samples, Alizarin Red S (40 mM, pH 4.2, 30 min) was used to visualize residual calcium. Native meniscus sections were stained with Alcian Blue (1% in 3% acetic acid, pH 2.5) to evaluate GAG distribution across zones. Slides were rinsed with DW and observed under an optical microscope.

#### Fourier transform infrared spectroscopy

Fourier transform infrared spectroscopy (FTIR) spectra of DMECM, RF and DMECM + RF hydrogels were recorded using an FTIR spectrometer (Nicolet iS50; Thermo Fisher Scientific, MA, USA) over the range of 4000–500 cm^−1^ to verify photo-crosslinking.

#### Rheology

Viscoelastic properties of DMECM-W hydrogels (2%, 2.5% and 3%) before and after photo-crosslinking were measured using a rheometer (Anton Paar, Graz, Austria). Storage (*G*′) and loss (*G*″) moduli were obtained within 1 0^−1^–10^2^ Hz and viscosity was determined over 10^−1^–10^3^ s^−1^ (*n* = 3).

#### Hydrogel infiltration into DBM scaffold

To assess infiltration, 2% (w/v) pre-DMECM-W hydrogel was applied to the top of cylindrical DBM scaffolds (8 mm × 3 mm) without photo-crosslinking. Images were captured at 0, 5 and 10 s using a digital camera.

#### Mechanical testing

The compressive modulus of native bone, DBM, and DBM + DMECM scaffolds was determined using a universal testing machine (Zwick Roell, Ulm, Germany) with a 5 kN load cell. Explanted DBM-only, DBM + DMECM-W and DBM + DMECM-I/M/O scaffolds after subcutaneous implantation were also tested. Samples were compressed at 10 mm/min up to 80% strain, and the modulus was calculated from the linear region of the stress–strain curve (*n* = 3).

#### Biochemical analysis

Double-stranded DNA (dsDNA), collagen and glycosaminoglycans (GAGs) were quantified for assessment of decellularization and ECM composition. Samples were digested in papain buffer (5 mM L-cysteine, 100 mM Na_2_HPO_4_, 5 mM EDTA, 125 μg/mL papain, 60°C, 12 h) for dsDNA and GAG assays, or solubilized in 0.1 M HCl with 0.1% pepsin (25°C, 24 h) for collagen. Quantification was performed using PicoGreen (Invitrogen), Biocolor B1000 (GAG) and Biocolor S1000 (collagen) kits and normalized to dry weight (*n* = 5).

#### Sodium dodecyl sulfate-polyacrylamide gel electrophoresis

Protein profiles of DMECM hydrogels were analyzed by sodium dodecyl sulfate-polyacrylamide gel electrophoresis (SDS–PAGE). Equal protein amounts (10 μg, Bradford assay) were mixed with Laemmli buffer (5% β-mercaptoethanol), heated (100°C, 10 min) and loaded onto 4–20% polyacrylamide gels (Bio-Rad). Gels were stained with Coomassie Brilliant Blue and imaged using a documentation system (Fusion SL2; Vilber).

#### Western blot

Fibrochondrogenic (COL1, TNC) and chondrogenic (COL2, ACAN, SOX9) proteins in DMECM hydrogels were analyzed by Western blot. Proteins (10 μg/lane) were separated by SDS–PAGE, transferred to PVDF membranes, blocked with 5% milk (1 h, Room Temperature) and incubated with primary antibodies (1:500, overnight, 4°C) followed by HRP-conjugated secondary antibodies (1:1000, 1 h, Room Temperature). Bands were visualized with ECL (Bio-Rad), post-stained with Coomassie Blue and quantified using ImageJ. Intensities were normalized to total lane density and expressed relative to DMECM-W (*n* = 5).

### Cellular behavior of DBM + DMECM scaffold

*In vitro* assays were performed to assess biocompatibility and biofunctionality of the DBM + DMECM scaffold. Human synovial membrane–derived MSCs were used to examine viability, adhesion, migration, proliferation and fibrochondrogenic differentiation, while human umbilical vein endothelial cells (HUVECs) were employed to evaluate zone-specific angiogenic responses, including viability, adhesion, migration, proliferation and tube formation.

#### Cell culture

MSCs were maintained in high-glucose DMEM (HyClone) supplemented with 10% FBS and 1% penicillin/streptomycin (Thermo Fisher Scientific) at 37°C, 5% CO_2_. Cells were subcultured at 80–90% confluence, and passage 3 was used. The identity and multipotency of MSCs were verified by immunophenotyping and trilineage differentiation prior to use (Supplementary Figure S1). HUVECs (CEFO, Seoul, Korea) were cultured in EGM-2 medium (CEFO) with 10% FBS and 1% penicillin/streptomycin under identical conditions, and passages 5–6 were used.

#### Cell adhesion assay

##### Cell seeding efficiency

Cell seeding efficiency (CSE) was evaluated as previously described [13]. MSCs (5 × 10^6^ cells/mL, 150 μL) were seeded onto DBM + DMECM scaffolds and incubated for 2 h at 37°C. After PBS washing, unattached and residual cells were collected by trypsinization and counted using a hemocytometer.


$$\text{CSE}\;\left(\%\right)=\left[1\;–\;\left({\text{cells}}_{u}/{\text{cells}}_{i}\right)\right]\times 100\%$$

where cells_*i*_ and cells_*u*_ represent the initially seeded and non-scaffold-attached cell numbers, respectively (*n* = 5).

##### Cell attachment assay

HUVEC attachment on DMECM coatings was assessed to determine the effect of zone-specific ECM composition. DMECM solutions (1 mg/mL) were coated on 96-well plates, incubated 2 h at 37°C, washed three times with PBS and seeded with HUVECs (5 × 10^3^ cells/well). After 2 h of culture, adherent cells were stained with calcein-AM (2 μg/mL; Invitrogen) for 30 min. Fluorescence intensity (Ex 494 nm, Em 520 nm) was measured using a microplate reader and expressed relative to control (*n* = 5).

#### Chemotactic migration assay

Chemotactic migration of MSCs toward scaffold-derived factors and HUVECs toward DMECM components was evaluated using Boyden chambers (ECM508; Millipore, USA). Serum-starved cells (1 × 10^5^ cells/mL) were seeded into the upper wells of 8 μm transwell inserts. The lower chambers contained scaffold-conditioned medium (1 mg/mL, 24 h) for MSCs or DMECM solutions (1 mg/mL; DMECM-W, I, M, O) for HUVECs. Serum-free medium served as control. After 4 h of incubation at 37°C, migrated cells were stained with trypan blue, eluted in extraction buffer (15 min, 25°C) and quantified spectrophotometrically at 560 nm (*n* = 5).

#### Live/dead assay

Cell viability of MSCs and HUVECs was assessed using a Live/Dead kit (L3224; Thermo Fisher Scientific). MSCs (1 × 10^6^ cells/mL) were seeded onto DBM + DMECM scaffolds and cultured for 24 h. HUVECs (5 × 10^3^ cells/well) were cultured in 96-well plates and treated with DMECM solutions (1 mg/mL; DMECM-W, I, M, O) for 24 h. After staining with PBS containing 2 μM calcein-AM and 4 μM ethidium homodimer-1 for 30 min at 25°C in the dark, cells were imaged using a confocal microscope (Leica Microsystems, Wetzlar, Germany) (*n* = 5).

#### Cell proliferation assay

Cell proliferation was assessed using a CCK-8 kit (CCK-3000; Dojindo, Japan). MSCs (1 × 10^6^ cells/mL) were seeded onto DBM + DMECM scaffolds and HUVECs (5 × 10^3^ cells/well) were cultured in 96-well plates treated with DMECM solutions (1 mg/mL; DMECM-W, I, M, O). Metabolic activity was measured on Day 1, 3 and 7. After medium removal, CCK-8 reagent (1:10 in fresh medium) was added and incubated for 2 h at 37°C. Absorbance was recorded at 450 nm using a microplate reader (*n* = 5).

#### RT-qPCR analysis

Fibrochondrogenic differentiation of MSCs on the DBM + DMECM scaffold was analyzed by RT-qPCR. MSCs (1 × 10^6^ cells/mL per scaffold) were pre-cultured in α-MEM with 10% FBS and 1% antibiotics for 24 h, then maintained in differentiation medium containing CTGF (100 ng/mL) and TGF-β3 (10 ng/mL) for 3 weeks. Total RNA was extracted (AccuPrep; Bioneer, Korea), reverse-transcribed (iScript; Bio-Rad, USA) and amplified using SYBR Green Master Mix (Roche, Switzerland) on a CFX96 system (Bio-Rad). Gene expression of COL1A1, TNC, COL2A1, ACAN and SOX9 was normalized to GAPDH and calculated by the 2^-^ΔΔCt method relative to DBM-only controls. Primer sequences are listed in Supplementary Table S1 (*n* = 3).

#### Tube formation analysis

Angiogenic potential of DMECM was evaluated by a tube formation assay using HUVECs. Matrigel (50 μL/well; Corning, NY, USA) was coated onto 96-well plates and polymerized at 37°C for 1 h. HUVECs (1 × 10^4^ cells/well) were seeded and treated with DMECM solutions (1 mg/mL; DMECM-W, I, M, O) in EGM-2 medium, while controls received EGM-2 only. After 24 h, tube-like structures were imaged under a light microscope, and quantitative analysis of loop number, branch points and total tube length was performed using ImageJ (NIH, Bethesda, MD, USA) (*n* = 5).

### Subcutaneous implantation of DBM + DMECM scaffold

#### Surgical procedure

MSC-seeded DBM + DMECM scaffolds (12 × 10 × 5 mm) were subcutaneously implanted into 4-week-old male nude mice (Koatech, Korea) under IACUC approval (No. 2023-0086). Twelve mice were used, each receiving one scaffold. Animals were anesthetized with Zoletil (1:10 in PBS), and a 1 cm dorsal incision was made to create a subcutaneous pocket. Sterile scaffolds were inserted, and incisions were closed with 4-0 sutures. Implants were harvested after 4 weeks and allocated for histological (*n* = 3), biochemical (collagen and GAG; *n* = 3) and mechanical (*n* = 3) analyses.

#### Histological evaluation

Explanted scaffolds were fixed in 10% formalin (72 h), paraffin-embedded and sectioned at 4 μm. Sections were stained with H&E for general morphology and Alcian Blue (1% in 3% acetic acid, pH 2.5) for GAG visualization. Images were captured under a light microscope, and vessel density was quantified by counting blood vessels in 10 random fields per region (inner, middle, outer) at ×50 magnification. The mean vessel count per scaffold was used for statistical analysis (*n* = 3).

#### Immunohistochemical analysis

Immunohistochemistry was performed to detect COL1, COL2, VEGF and CD31. Sections were deparaffinized, rehydrated and treated with 3% hydrogen peroxide, followed by blocking with 5% BSA for 1 h. Primary antibodies (1:100, Abcam) were applied overnight at 4°C, followed by HRP-conjugated secondary antibodies (1:100, 1 h, room temperature). Signals were visualized with DAB and counterstained with hematoxylin. For quantification, five random fields per region were analyzed, and mean staining intensity per scaffold was calculated using ImageJ (*n* = 3).

#### Biochemical analysis

Explanted scaffolds were analyzed for collagen and GAG contents using the same biochemical protocols described in Section Biochemical analysis (*n* = 3).

### Statistical analysis

All experiments were performed in triplicate unless otherwise specified. Data were analyzed using GraphPad Prism 8 (GraphPad Software, La Jolla, CA, USA). Differences between two groups were evaluated by Student’s *t*-test, and multiple group comparisons by one-way ANOVA followed by Tukey’s *post hoc* test. Results are presented as mean ± standard deviation (SD). Statistical significance was set at *P *< 0.05 (*), *P *< 0.01 (@) and *P *< 0.001 (#).

## Results

### Physicochemical characterization of DBM scaffolds

**Figure 1:**
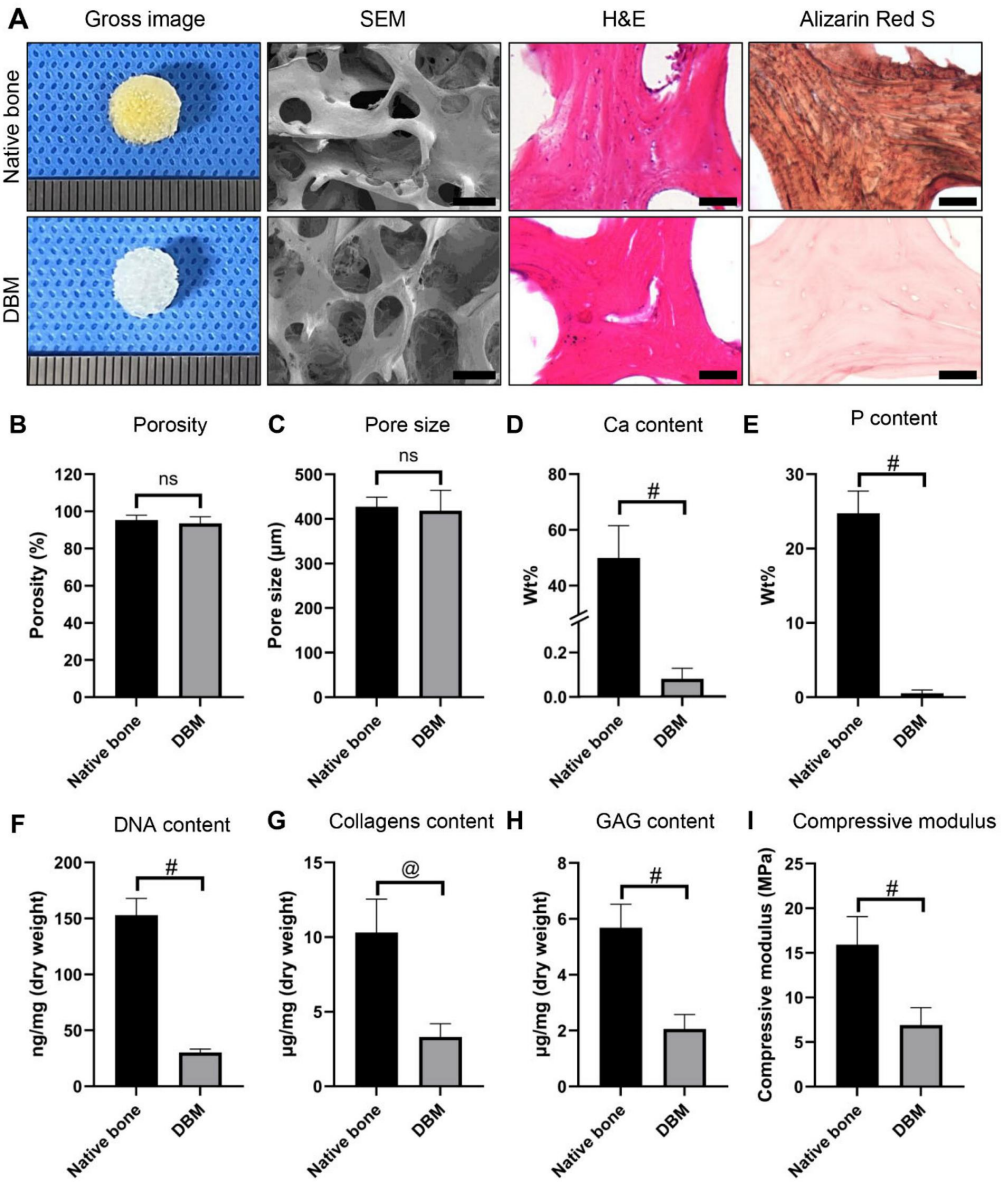
Physicochemical characterization of native bone and DBM. (**A**) Representative gross, SEM, H&E and Alizarin Red S images of native bone and DBM. (**B**, **C**) Porosity and pore size analysis. (**D**, **E**) Elemental analysis of calcium (Ca) and phosphorus (P) by EDX. (**F**–**H**) Quantification of residual DNA, collagen and GAG contents. (**I**) Measurement of compressive modulus of native bone and DBM. Data are expressed as mean ± SD (*n* = 6). Statistical significance was assessed using an unpaired *t*-test (*^@^P *< 0.01, *^#^P *< 0.001). Scale bars: 1 mm (gross), 500 µm (SEM) and 200 µm (H&E).

The DBM scaffold retained a porous, sponge-like architecture that resembled native trabecular bone. Representative micrographs confirmed preservation of the collagenous framework, complete cellular removal and effective decalcification (Figure 1A). Quantitative analysis revealed high porosity (93.5 ± 3.6%) and a mean pore size of 418.5 ± 45.8 μm, indicating favorable conditions for nutrient diffusion and cell infiltration (Figure 1B and C). Residual DNA content (30.2 ± 3.0 ng/mg) was below the accepted decellularization threshold (Figure 1F), while EDX spectra confirmed marked reductions in calcium and phosphorus peaks (Figure 1D and E). Collagen and GAG levels decreased from 10.3 ± 2.2–3.3 ± 0.9 μg/mg and from 5.7 ± 0.8–2.1 ± 0.5 μg/mg, respectively (Figure 1G and H). The compressive modulus of DBM (6.9 ± 2.0 MPa) was approximately 57% lower than that of native bone (15.9 ± 3.1 MPa), reflecting partial stiffness reduction while maintaining structural integrity (Figure 1I). These findings demonstrate that the DBM scaffold maintains a stable, decellularized collagen framework appropriate for DMECM loading.

### Biochemical characterization of zone-specific DMECM

**Figure 2:**
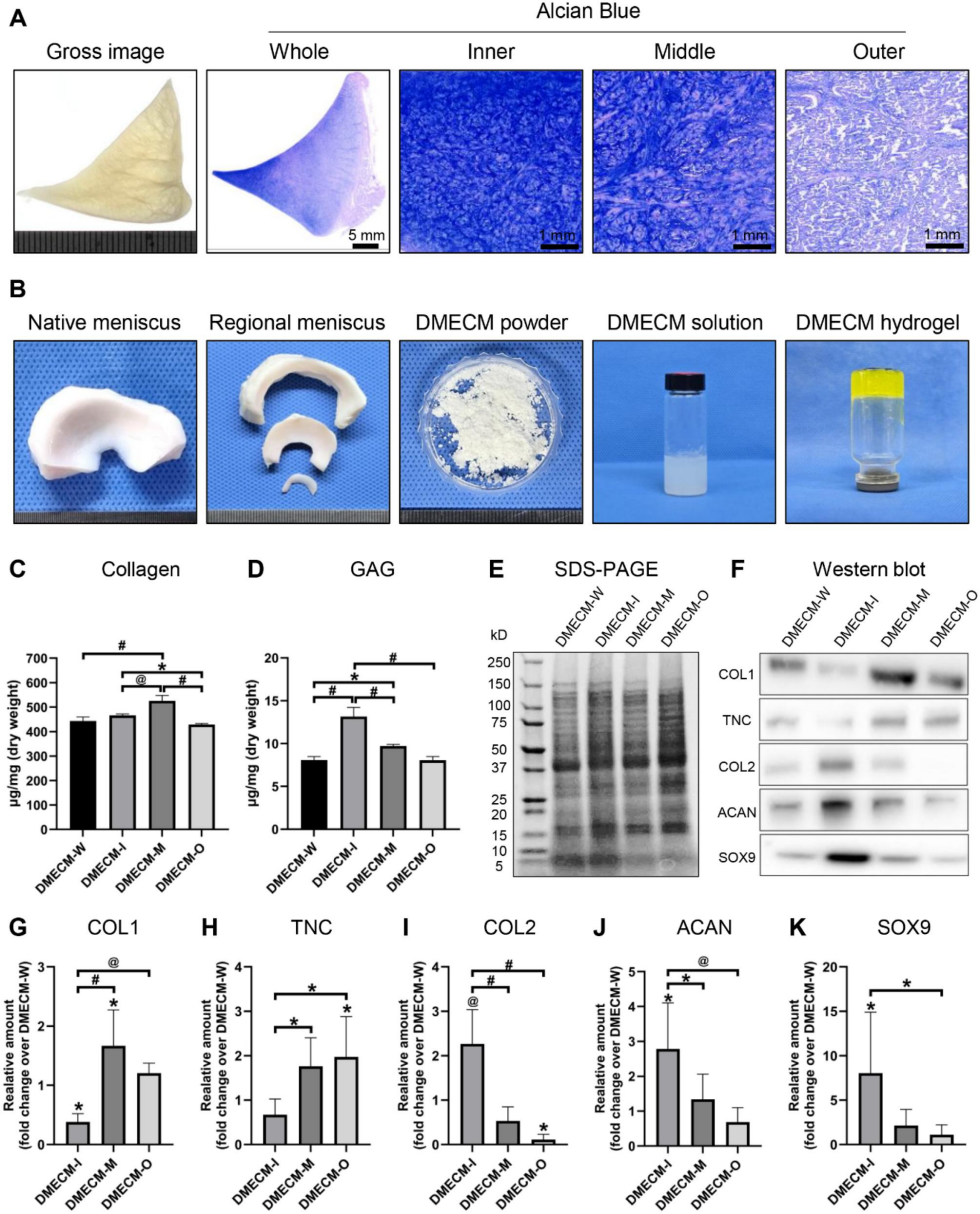
Biochemical characterization of zone-specific decellularized meniscus ECM (DMECM) hydrogels. (**A**) Cross-sectional and Alcian Blue–stained images of the native meniscus showing region-dependent fibrous architecture and GAG intensity. (**B**) Sequential gross images of DMECM preparation: native meniscus, regionally dissected (inner, middle and outer) segments, decellularized DMECM powder, pepsin-digested DMECM solution and DMECM hydrogel prepared via riboflavin-mediated photo-crosslinking. (**C**, **D**) Collagen and GAG quantification. (**E**) SDS-PAGE of total ECM protein profiles. (**F**–**K**) Western blot analysis of COL1, TNC, COL2, ACAN and SOX9, with densitometric quantification normalized to DMECM-W. Data are expressed as mean ± SD (*n* = 5). Statistical significance was evaluated using one-way ANOVA with Tukey’s multiple comparisons test (*P < 0.05, *^@^P *< 0.01, *^#^P *< 0.001).

Cross-sectional imaging of the native meniscus revealed dense collagen bundles extending from the outer root toward the middle zone (Figure 2A). Alcian Blue staining showed a progressive increase in GAG intensity from the outer to inner regions, highlighting region-dependent biochemical heterogeneity. Sequential gross images illustrated each preparation step—from native meniscus and regionally dissected tissues to decellularized DMECM powder, pepsin-digested solution and riboflavin–crosslinked DMECM hydrogel (Figure 2B).

To assess biochemical zonation, collagen and GAG contents were quantified in DMECM-W, -I, -M and -O hydrogels (Figure 2C and D). Collagen was most abundant in DMECM-M, followed by DMECM-I, whereas DMECM-W and DMECM-O contained lower levels. GAG content was highest in DMECM-I, decreased in DMECM-M and was lowest in DMECM-W and DMECM-O. SDS–PAGE confirmed preservation of multiple ECM protein bands across all groups (Figure 2E). Western blot analysis further demonstrated zone-specific protein expression: COL1 and TNC predominated in the outer and middle regions, while COL2, ACAN and SOX9 were enriched in DMECM-I and decreased toward the outer regions (Figure 2F–K).

Taken together, these findings demonstrate that each DMECM hydrogel preserves region-specific biochemical characteristics reflective of its native meniscal zone.

### Optimization of DMECM hydrogel

**Figure 3:**
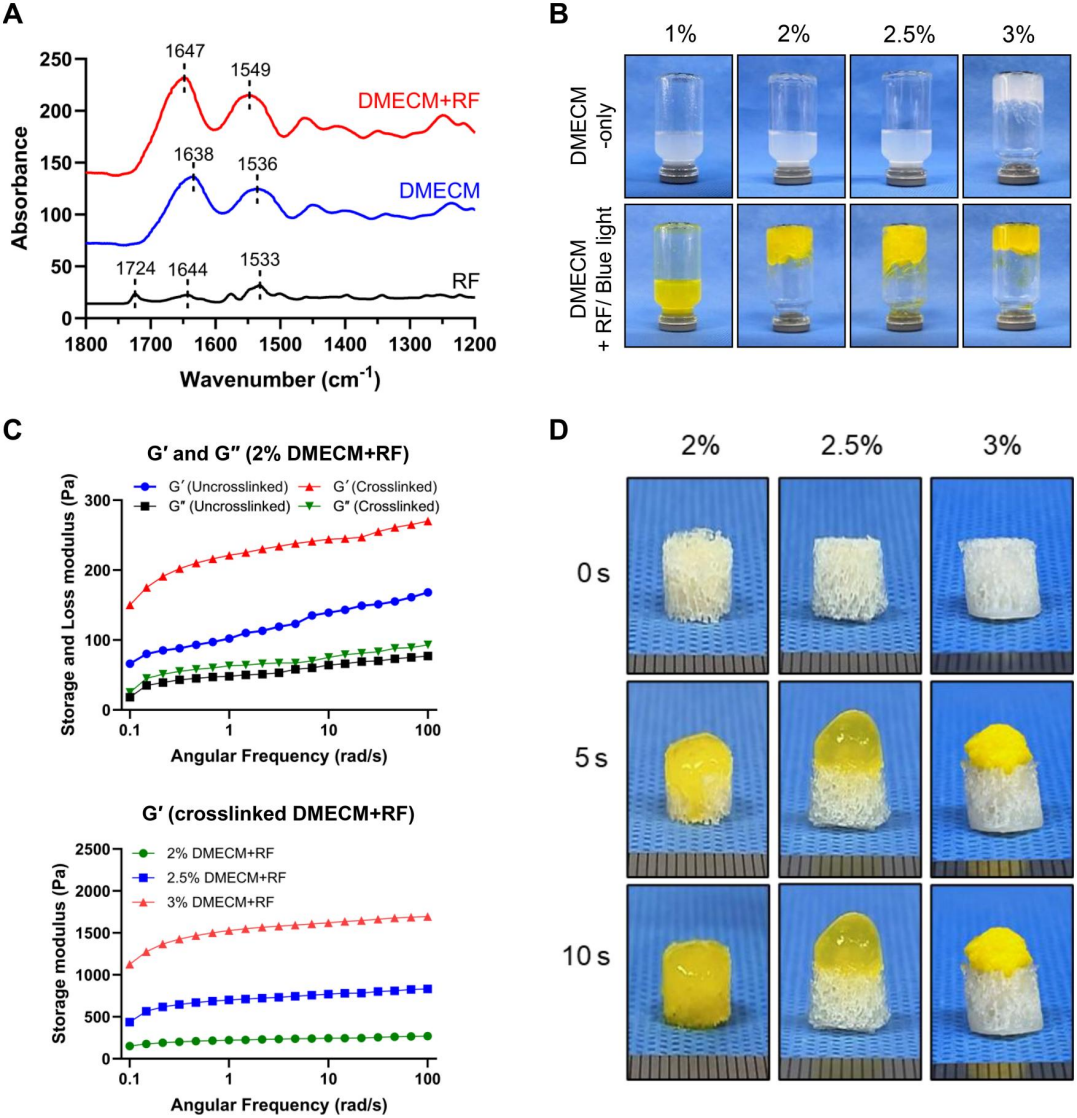
Optimization of DMECM hydrogel properties for DBM scaffold application. (**A**) FTIR spectra of DMECM, RF and DMECM + RF showing characteristic amide I and II bands (1800–1200 cm^−1^). (**B**) Gross appearance of 1–3% (w/v) DMECM hydrogels after photo-crosslinking. (**C**) Frequency sweep analysis of 2% DMECM + RF hydrogels before and after photo-crosslinking (upper) and storage modulus comparison among 2%, 2.5% and 3% DMECM + RF hydrogels (lower). (**D**) Infiltration behavior of 2–3% DMECM hydrogels into DBM scaffolds at 0, 5 and 10 s.

To determine the optimal DMECM concentration, 1%, 2%, 2.5% and 3% (w/v) DMECM-W solutions were evaluated for gelation, rheological behavior and scaffold infiltration (Figure 3). FTIR spectra confirmed photo-crosslinking between DMECM and RF. Characteristic RF peaks were observed at 1724 cm^−1^ (C=O stretching) and 1644–1533 cm^−1^ (aromatic C=C stretching). In the DMECM + RF group, amide I and II bands shifted from 1638/1536 cm^−1^ to 1647/1549 cm^−1^ with increased intensity, confirming covalent bonding between ECM proteins and RF (Figure 3A).

Visually, 1% DMECM remained flowable, whereas 2% and 2.5% formed stable gels and 3% was excessively viscous (Figure 3B). Rheological analysis showed that 2% DMECM + RF exhibited significantly higher storage (*G*′) and loss (*G*″) moduli after photo-crosslinking, confirming network stabilization (Figure 3C, upper). Moreover, *G*′ values increased in a concentration-dependent manner among crosslinked 2%, 2.5% and 3% hydrogels (Figure 3C, lower). Infiltration assays demonstrated that only 2% DMECM completely penetrated the DBM scaffold within 10 s, whereas higher concentrations remained superficial (Figure 3D).

Collectively, these results establish 2% DMECM as the optimal formulation, providing balanced gelation, tunable stiffness and excellent scaffold compatibility.

### Fabrication and optimization of zone-specific DBM + DMECM composite scaffolds

**Figure 4:**
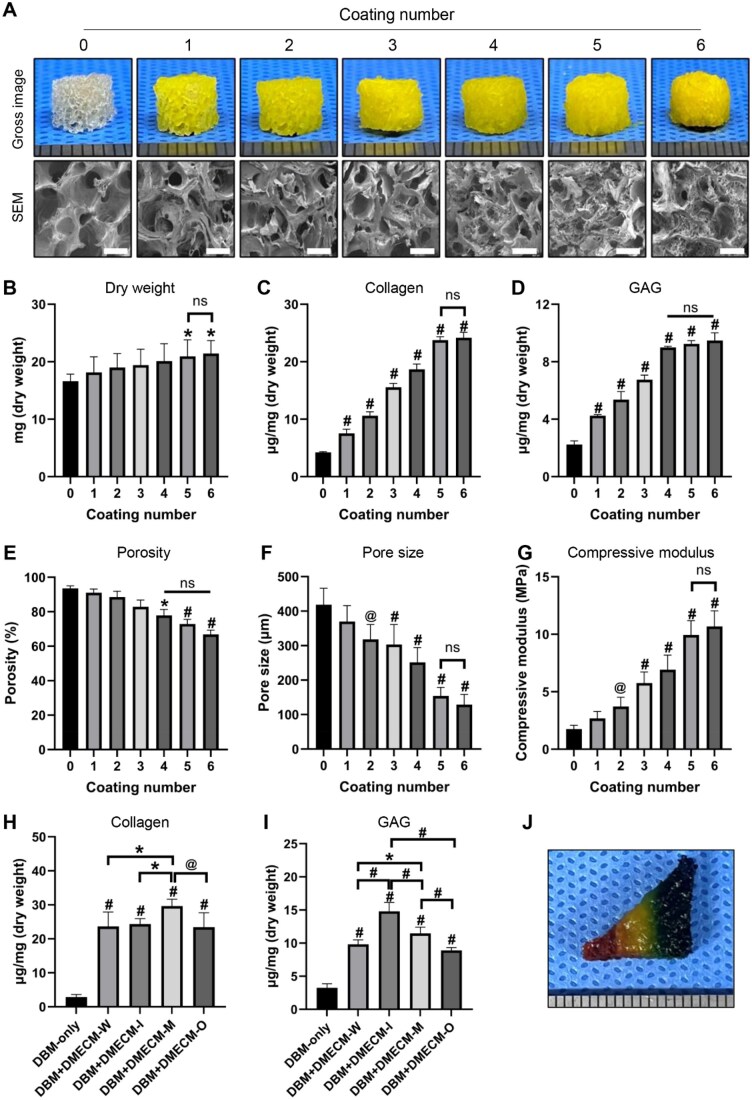
Optimization and fabrication of zone-specific DBM + DMECM composite scaffolds. (**A**) Gross and SEM images of DBM scaffolds coated with 0–6 cycles of DMECM-W. (**B**–**G**) Dry weight, collagen and GAG contents, porosity, pore size and compressive modulus quantification. (**H**, **I**) Collagen and GAG analysis in DBM scaffolds coated with zone-specific DMECM-I, -M and -O hydrogels. (**J**) Gross image of a triangular prism–shaped DBM scaffold loaded with zone-specific hydrogels: DMECM-I, DMECM-M and DMECM-O. Data are expressed as mean ± SD (*n* = 6). Statistical significance was determined using one-way ANOVA with Tukey’s multiple comparisons test (*P < 0.05, *^@^P *< 0.01, *^#^P *< 0.001). Scale bars: 1 mm (gross), 500 µm (SEM).

To optimize hydrogel incorporation, DBM scaffolds were subjected to 0–6 coating cycles of 2% DMECM-W followed by blue-light crosslinking. Gross and SEM observations revealed progressive hydrogel deposition and gradual pore filling with increasing coating cycles (Figure 4A). Correspondingly, dry weight increased in a cycle-dependent manner (Figure 4B). Collagen and GAG levels rose proportionally up to five coatings, with no further gain at six (Figure 4C and D). Porosity and pore size decreased with additional coatings but remained above functional thresholds (porosity >70%, pore size >150 μm) up to five cycles, falling below these values thereafter (Figure 4E and F). The compressive modulus increased concurrently, indicating enhanced structural reinforcement (Figure 4G). Thus, five coating cycles were identified as optimal, achieving balanced matrix enrichment, mechanical integrity and pore interconnectivity.

Using this optimized protocol, zone-specific DMECM hydrogels (DMECM-I, -M and -O) were applied to DBM scaffolds to reproduce the biochemical heterogeneity of native meniscus tissue. Collagen content was highest in DBM + DMECM-M, whereas GAG content peaked in DBM + DMECM-I, reflecting the intrinsic zonal composition of meniscal ECM (Figure 4H and I). Finally, triangular prism–shaped DBM scaffolds were sequentially coated with red, yellow and blue hydrogels corresponding to DMECM-I, -M and -O, respectively, demonstrating the feasibility of constructing a zone-specific composite scaffold that spatially mimics the native meniscal structure (Figure 4J).

Overall, these findings indicate that the optimized five-cycle coating enabled the fabrication of a zone-specific DBM + DMECM composite scaffold that spatially reproduces the native meniscal zonation.

### Effect of DBM + DMECM scaffolds on MSC behavior

**Figure 5:**
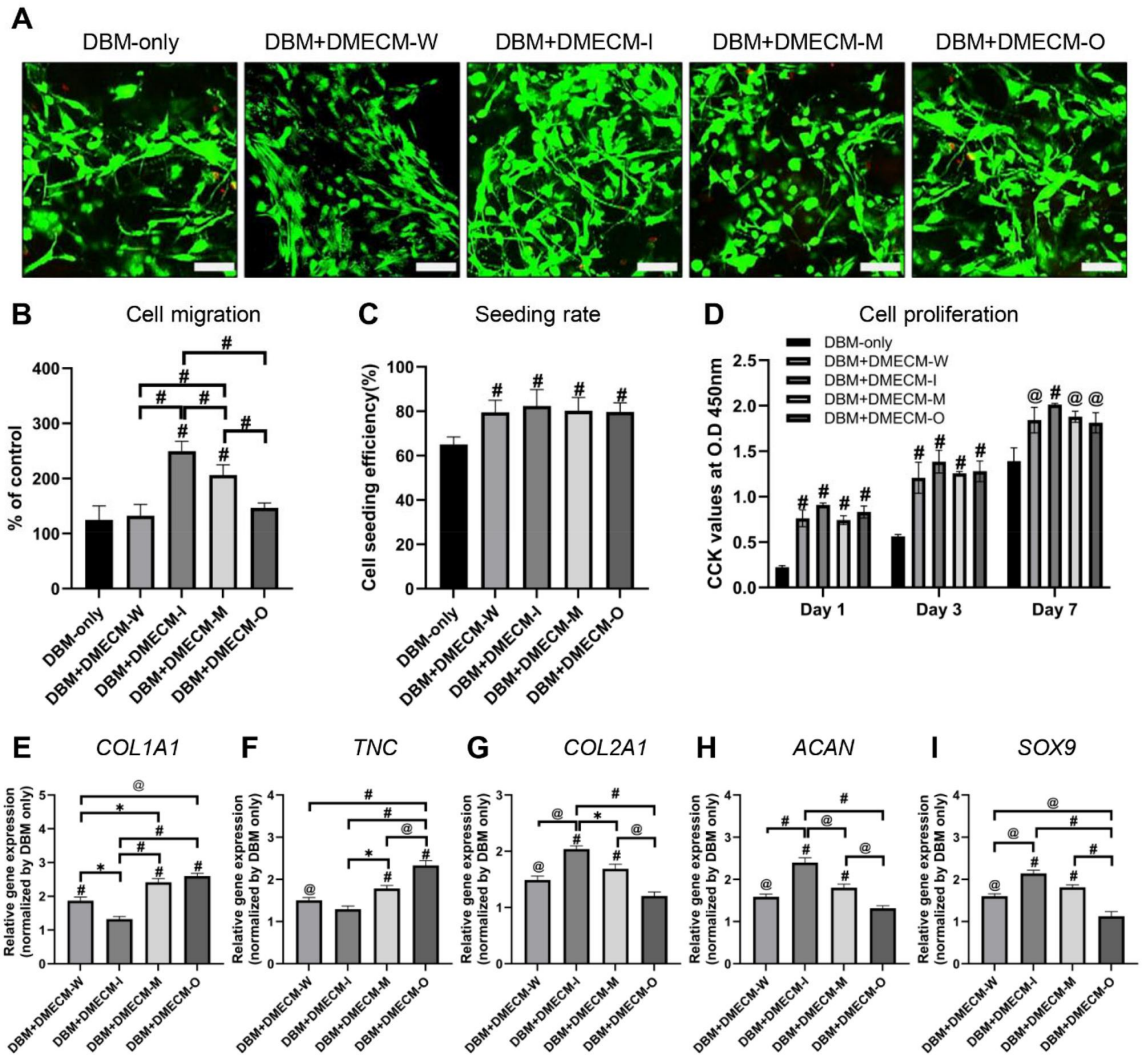
*In vitro* evaluation of MSC behavior on zone-specific DBM + DMECM scaffolds. (**A**) Live/dead staining of MSCs after 24 h of culture. (**B**–**D**) Chemotactic migration, CSE and proliferation after 1, 3 and 7 days. (**E**–**I**) RT-qPCR analysis of COL1, TNC, COL2, ACAN and SOX9 gene expression after 3 weeks of differentiation. Data are expressed as mean ± SD (*n* = 5 for migration, seeding and proliferation; *n* = 3 for gene expression). Statistical significance was evaluated using one-way ANOVA with Tukey’s multiple comparisons test (*P < 0.05, *^@^P *< 0.01, *^#^P *< 0.001). Scale bars: 100 µm.

To assess *in vitro* biocompatibility and cellular responses, MSCs were seeded onto DBM-only and zone-specific DBM + DMECM composite scaffolds. Live/Dead staining after 24 h showed predominantly viable (green) and minimal dead (red) cells across all groups, confirming noncytotoxicity (Figure 5A). Chemotactic migration was significantly enhanced in the DBM + DMECM-I group, followed by DBM + DMECM-M, whereas DBM + DMECM-W and DBM + DMECM-O showed no significant difference from DBM-only (Figure 5B). CSE was markedly higher in all DMECM-coated scaffolds, with the DBM + DMECM-I group exhibiting the highest rate (Figure 5C). CCK-8 assay demonstrated time-dependent proliferation in all groups, with significantly greater metabolic activity in all DMECM-coated scaffolds compared with DBM-only at Days 1, 3 and 7 (Figure 5D).

After 3 weeks of fibrochondrogenic induction, RT-qPCR analysis revealed that DBM + DMECM-I showed the highest expression of chondrogenic markers (COL2A1, ACAN, SOX9), whereas DBM + DMECM-O exhibited the strongest fibrogenic marker expression (COL1A1, TNC) (Figure 5E–I). The DBM + DMECM-M group displayed intermediate expression patterns, consistent with its transitional biochemical composition.

Taken together, these findings demonstrate that zone-specific DMECM hydrogels distinctly modulate MSC adhesion, migration and proliferation according to regional composition and selectively promote region-dependent fibrochondrogenic differentiation *in vitro*.

### *In vivo* fibrochondrogenic tissue formation induced by DBM + DMECM scaffolds

**Figure 6:**
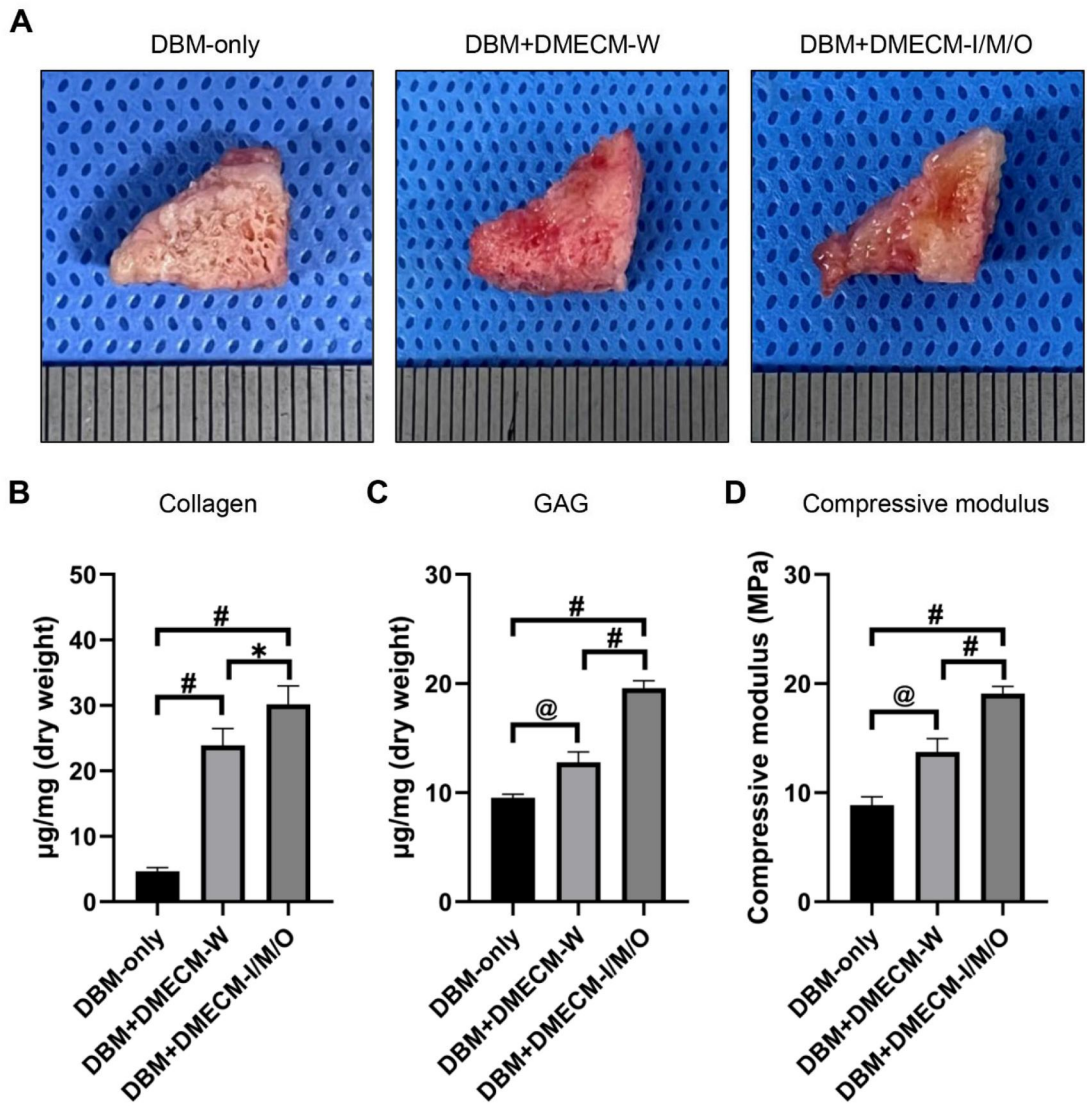
*In vivo* evaluation of MSC-seeded DBM + DMECM scaffolds. (**A**) Gross images of MSC-seeded scaffolds retrieved from nude mice at 4 weeks post-implantation. (**B**–**D**) Collagen content, GAG content and compressive modulus quantification. Data are expressed as mean ± SD (*n* = 3). Statistical significance was evaluated using one-way ANOVA with Tukey’s multiple comparisons test (*P < 0.05, *^@^P *< 0.01, *^#^P *< 0.001). Scale bars: 1 mm.

To assess *in vivo* tissue remodeling, MSC-seeded DBM scaffolds were subcutaneously implanted into nude mice and harvested after 4 weeks. Gross inspection showed intact, well-integrated constructs without signs of inflammation or degradation. Zone-specific DBM + DMECM scaffolds appeared more hydrated and compact than DBM-only or DBM + DMECM-W counterparts (Figure 6A). Biochemical analyses revealed significantly higher collagen and GAG contents, along with increased compressive modulus, in all DMECM-coated scaffolds compared with DBM-only controls. Among these, the zone-specific DBM + DMECM-I/M/O group exhibited the highest values across all parameters, indicating enhanced ECM deposition and mechanical reinforcement (Figure 6B–D).

**Figure 7:**
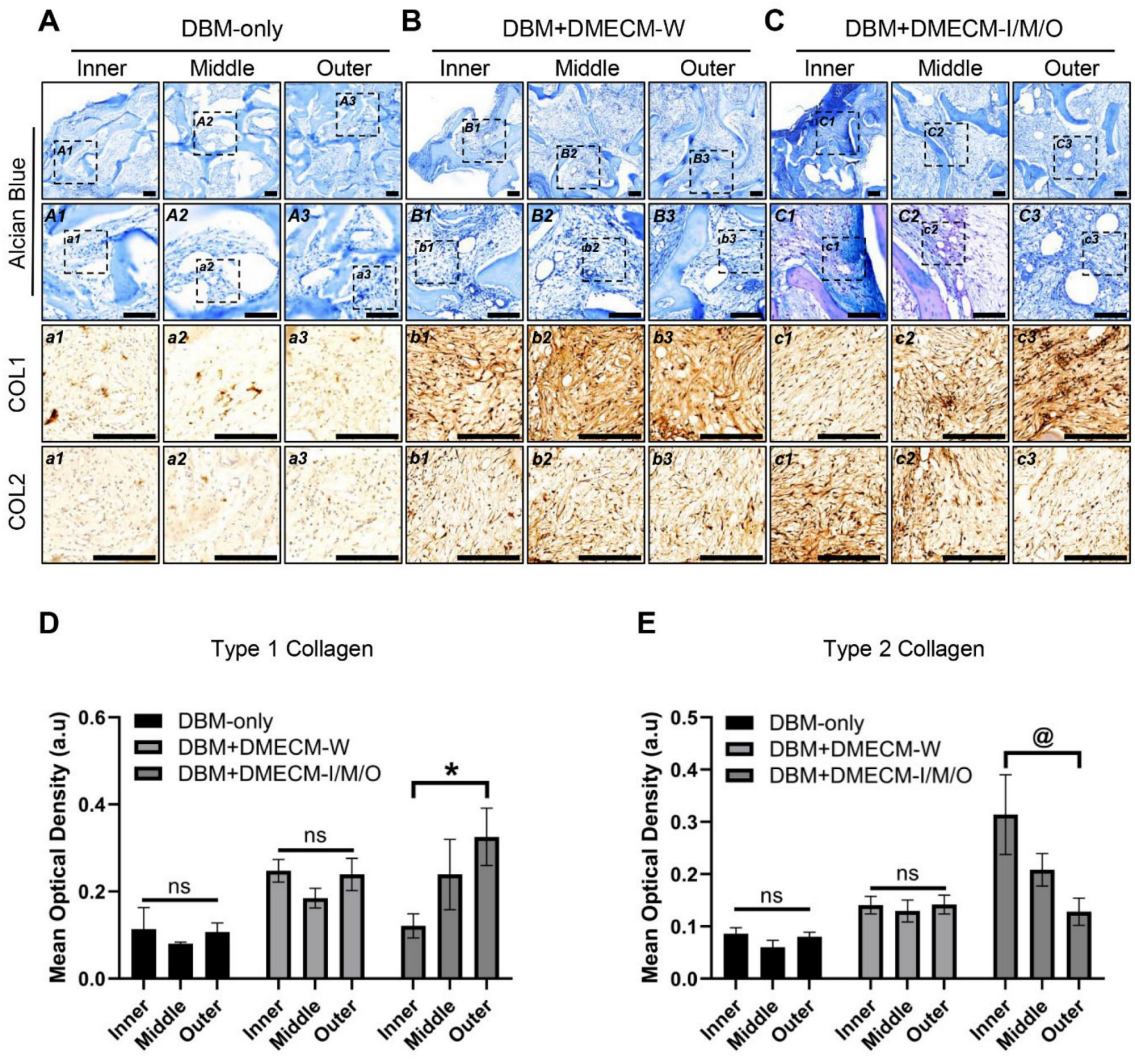
Histological evaluation of MSC-seeded DBM + DMECM scaffolds. (**A**–**C**) Low- and high-magnification Alcian Blue staining of retrieved constructs showing GAG deposition and matrix formation at 4 weeks post-implantation, with corresponding high-magnification regions immunostained for COL1 and COL2. (**D**, **E**) Quantitative analysis of COL1 and COL2 immunostaining intensity. Data are expressed as mean ± SD (*n* = 3). Statistical significance was evaluated using one-way ANOVA with Tukey’s multiple comparisons test (*P < 0.05, *^@^P *< 0.01, *^#^P *< 0.001). Scale bars: 100 µm. Whole-slide panoramic Alcian Blue images illustrating spatial ECM distribution are provided in Supplementary Figure S3.

Histological and immunohistochemical (IHC) analyses further elucidated regional matrix remodeling (Figure 7). DBM-only scaffolds showed limited, disorganized ECM deposition, whereas DBM + DMECM-W exhibited moderate but nonspecific matrix formation. In contrast, zone-specific DBM + DMECM-I/M/O scaffolds displayed pronounced spatial heterogeneity, with Alcian Blue and COL2 staining strongest in the inner region and COL1 predominating in the outer region. Quantitative IHC confirmed these gradients, showing an inverse correlation between COL1 and COL2 expression across zones.

Collectively, these results demonstrate that spatially patterned DMECM coatings effectively direct region-specific fibrochondrogenic remodeling *in vivo*, recapitulating the native meniscal zonation and structural hierarchy.

### *In vivo* neovascularization induced by DBM + DMECM scaffolds

**Figure 8:**
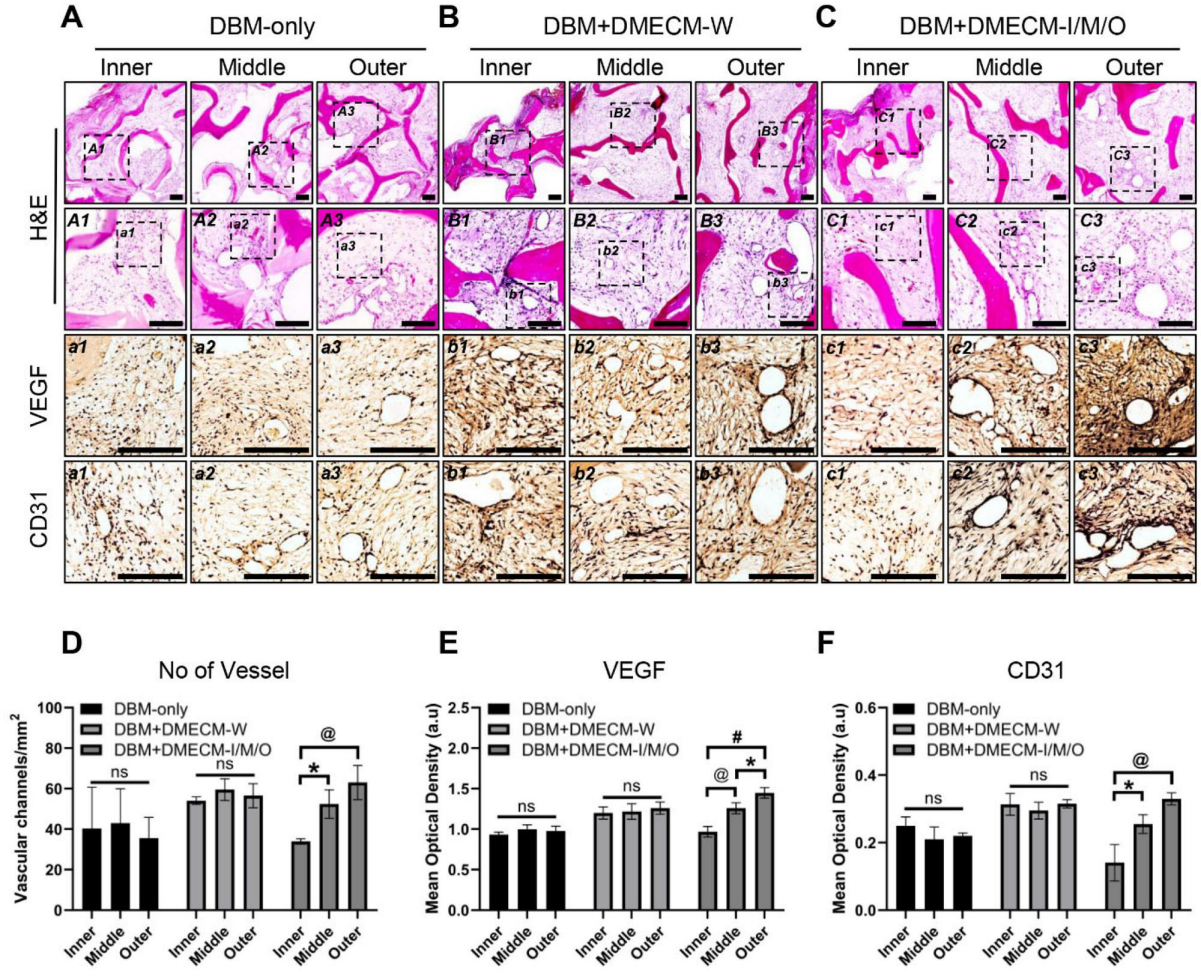
Evaluation of neovascularization in MSC-seeded DBM + DMECM scaffolds. (**A**–**C**) Low- and high-magnification H&E staining of retrieved constructs showing tissue integration and neovascularization at 4 weeks post-implantation, with corresponding high-magnification regions immunostained for VEGF and CD31. (**D**) Quantification of vessel density from H&E images. (**E**, **F**) Quantification of VEGF and CD31 staining intensity. Data are expressed as mean ± SD (*n* = 3). Statistical significance was evaluated using one-way ANOVA with Tukey’s multiple comparisons test (*P < 0.05, *^@^P *< 0.01, *^#^P *< 0.001). Scale bars: 100 µm. Whole-slide panoramic H&E images illustrating spatial tissue distribution are provided in Supplementary Figure S3.

After 4 weeks of subcutaneous implantation, H&E staining revealed vascular infiltration throughout the DBM framework in all groups (Figure 8A–C). Notably, the DBM + DMECM-I/M/O scaffolds exhibited a distinct zonal vascular pattern, characterized by sparse microvessels in the inner region and progressively increased vascular density toward the outer region. In contrast, the DBM-only and DBM + DMECM-W scaffolds displayed uniform vascular distribution without regional differences.

Total vessel counts integrated across all regions were higher in both DMECM-coated groups compared with DBM-only scaffolds, as shown in Supplementary Figure S2. When vessel density was analyzed on a region-by-region basis, however, the DBM-only and DBM + DMECM-W scaffolds showed comparable values across zones, whereas the DBM + DMECM-I/M/O scaffolds exhibited a clear inner-to-outer gradient (Figure 8D). Immunohistochemical staining for VEGF and CD31 (Figure 8E and F) further supported this observation, revealing parallel increases in angiogenic marker expression toward the outer zone.

Together, these data indicate that spatially organized DMECM coatings reestablish a region-dependent angiogenic gradient reminiscent of the native meniscus.

### *In vitro* validation of zone-specific DMECM effects on HUVEC angiogenic response

**Figure 9:**
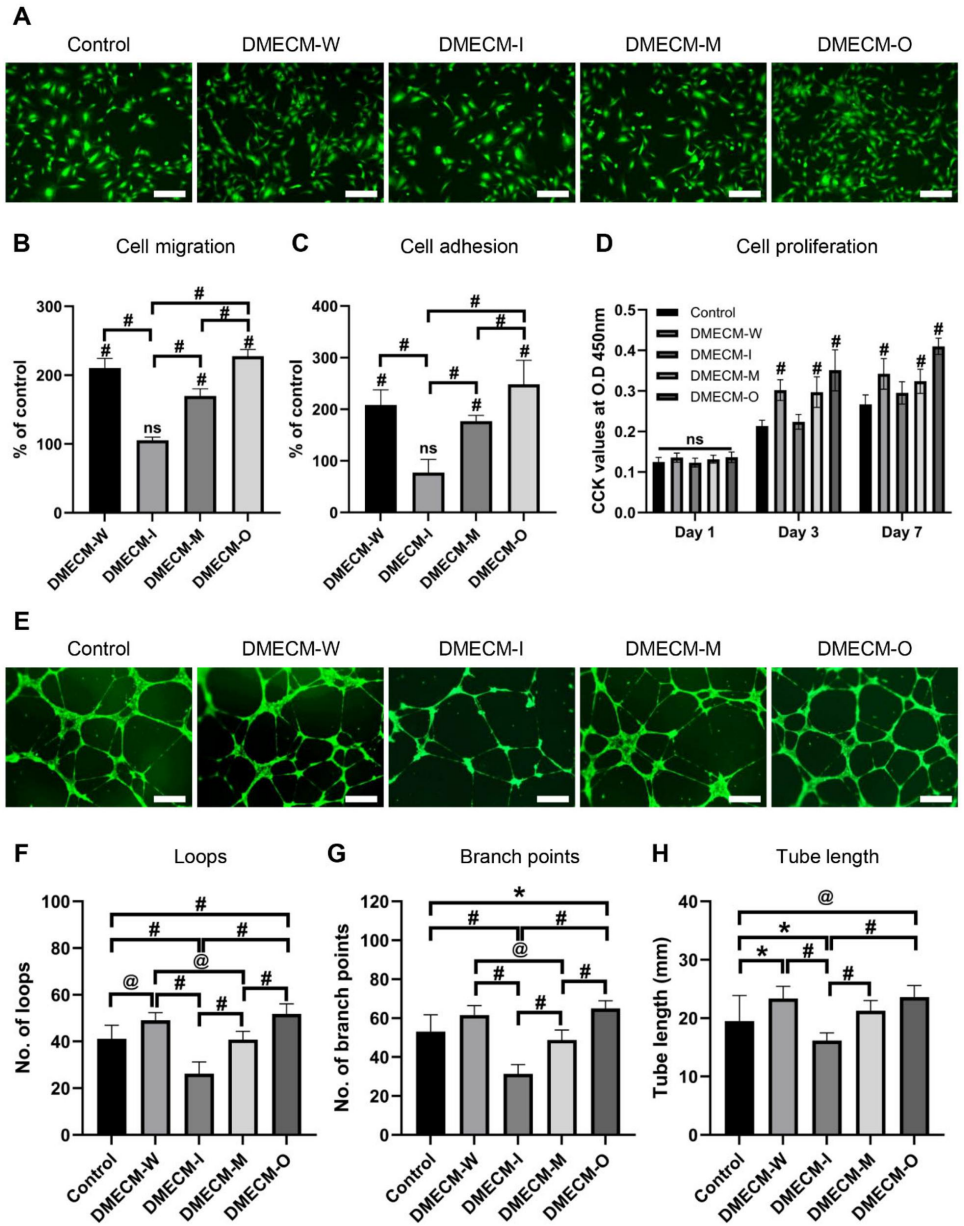
*In vitro* validation of angiogenic activity of zone-specific DMECM. (**A**) Live/dead fluorescence images of HUVECs cultured with DMECM solutions (1 mg/mL, 24 h, noncrosslinked). (**B**) Chemotactic migration of HUVECs toward DMECM solutions analyzed by transwell assay. (**C**) Quantitative analysis of HUVEC adhesion after 2 h incubation on DMECM-coated substrates. (**D**) Time-dependent proliferation of HUVECs after 1, 3 and 7 days. (**E**) Representative tube formation images of HUVECs cultured on matrigel treated with DMECM solutions. (**F**–**H**) Quantitative analysis of tube formation parameters showing loop number, branch points and total tube length. Data are expressed as mean ± SD (*n* = 5). Statistical significance was evaluated using one-way ANOVA with Tukey’s multiple comparisons test (*P < 0.05, *^@^P *< 0.01, *^#^P *< 0.001). Scale bars: 500 µm.

To determine whether this zone-dependent vascular pattern originated from intrinsic DMECM cues, we next examined the angiogenic behavior of HUVECs cultured with isolated DMECM-I, DMECM-M and DMECM-O solutions (Figure 9).

Live/Dead staining confirmed high viability across all groups (Figure 9A). Migration and adhesion assays demonstrated minimal endothelial activation in response to DMECM-I, whereas DMECM-M, DMECM-W and DMECM-O progressively enhanced chemotaxis and adhesion (Figure 9B and C). Proliferation assays revealed a similar trend, with DMECM-O inducing the highest metabolic activity (Figure 9D).

In tube formation assays, DMECM-O promoted extensive capillary-like network development, while DMECM-I produced the lowest loop numbers, branching points and tube lengths, consistent with an anti-angiogenic phenotype (Figure 9E–H).

Collectively, these findings validate that the region-dependent angiogenic behavior observed *in vivo* arises from intrinsic biochemical differences within the DMECM compartments, reflecting the natural avascular-to-vascular hierarchy of the meniscus.

## Discussion

### Major findings

This study developed a triphasic DBM + DMECM scaffold that spatially reproduces the native biochemical and vascular zonation of the meniscus. By combining the mechanical stability of DBM with region-specific ECM hydrogels, the construct directed zone-dependent MSC differentiation, supported organized ECM remodeling *in vivo* and established a physiological angiogenic gradient. Endothelial assays confirmed that these vascular patterns arise from intrinsic DMECM cues. Together, these findings demonstrate a biomimetic strategy capable of coordinating fibrocartilaginous and angiogenic processes essential for functional meniscus regeneration. Compared with prior scaffolds based on homogeneous ECMs, synthetic polymers or nonzonal biomaterials, our construct uniquely integrates structural and biochemical zonation within a single platform, providing a robust foundation for functional meniscal regeneration and broader gradient-guided tissue engineering applications.

### Zone-specific fibrochondrogenic differentiation regulated by DMECM

The DMECM hydrogels preserved the intrinsic biochemical heterogeneity of the native meniscus (Figure 2). Our previous proteomic profiling [6] showed that DMECM-I was enriched in cartilage-associated proteins, such as ACAN, HAPLN1, MATN (primarily MATN1 and MATN3), CILP (CILP and CILP2), TGFBI and COL2, forming a GAG-rich microenvironment. DMECM-M exhibited a transitional ECM composition dominated by small leucine-rich proteoglycans (SLRPs), including FMOD, DCN, CHAD, BGN, PRELP and OGN, whereas DMECM-O contained abundant fibrous ECM proteins, including COL1, COL3, TNXB, TNC, VCAN, ASPN and LOX. This zonal ECM composition reflects the spatial organization of native fibrocartilage and corresponds well with the zone-specific MSC gene expression patterns observed in Figure 5E–I and the ECM remodeling profiles shown in Figure 7.

Beyond compositional differences, evidence from existing mechanobiology studies suggests that such zonal ECM features may provide distinct biophysical cues that influence MSC behavior. The GAG-rich DMECM-I, containing abundant ACAN and HAPLN1, may enhance hydration, osmotic swelling pressure and pericellular micromechanics [14]. These biophysical cues align with findings that GAG-rich matrices—particularly those incorporating chondroitin sulfate—augment osmotic and compressive signals in 3D environments and consequently promote SOX9-mediated chondrogenesis [15, 16]. Notably, this mechanistic trend is consistent with our observation that COL2, ACAN and SOX9 gene expression were selectively upregulated in the DBM + DMECM-I scaffold (Figure 5E–I). These combined biophysical and transcriptional patterns likely contribute to the pronounced chondrogenic responses observed in the DMECM-I compartment.

The SLRP-enriched DMECM-M suggests a transitional ECM capable of modulating collagen fibrillogenesis and intermediate-level matrix organization [14]. Such properties align with the mixed COL1/COL2 phenotype detected in the middle region and resemble the structural characteristics of the native meniscal intermediate zone, which bridges the cartilage-like inner region and the fibrous outer region [6, 17]. This may also explain the balanced fibrochondrogenic marker expression observed in Figure 5E–I.

DMECM-O, enriched in COL1/3, TNXB, TNC, VCAN, ASPN and LOX, likely provides a tensile and adhesion-rich environment [7, 17]. Fibrous ECM constituents can enhance integrin engagement, focal adhesion formation and actomyosin tension, promoting nuclear YAP activity [14, 18]. The presence of LOX further suggests increased collagen crosslinking and tensile stiffness, consistent with the fibrous remodeling and TNC/COL1 deposition patterns observed in Figure 7.

Collectively, these findings suggest that DMECM-I/M/O provide distinct biochemical and potentially mechanobiological microenvironments that guide zone-specific MSC differentiation. DMECM-I may generate a GAG-rich, compressive niche; DMECM-M a transitional fibril-modulating niche; and DMECM-O, a tensile, adhesion-mediated niche. While specific mechanotransduction pathways were not examined directly, the integration of proteomic data with established ECM-mechanosensing principles provides a coherent framework for interpreting the zone-dependent fibrochondrogenic outcomes observed in this study.

### Zone-dependent angiogenic regulation mediated by DMECM and its significance in meniscal regeneration

The DBM + DMECM-I/M/O scaffolds exhibited a clear zone-dependent vascular hierarchy *in vivo* (Figure 8), with minimal vessel infiltration in the inner region, moderate levels in the middle region and greater vascularization in the outer zone. A similar trend appeared *in vitro* (Figure 9): DMECM-I suppressed endothelial tube formation, DMECM-M produced an intermediate response and DMECM-O permitted sprouting. Together with the increased total vessel infiltration observed in DMECM-coated scaffolds (Supplementary Figure S2), these findings demonstrate that both the magnitude and spatial distribution of angiogenic responses are governed by compartment-specific ECM cues.

The meniscus shows a defined transition from a vascularized periphery to an avascular inner white–white zone [4], where a dense, GAG-rich fibrocartilaginous niche restricts endothelial ingress [19]. Although its anti-angiogenic composition is not fully characterized, cartilage tissues maintain avascularity through regulators such as chondroitin sulfate, chondromodulin-1, endostatin and thrombospondins [20, 21], suggesting that similar biochemical principles may operate in fibrocartilaginous tissues. Supporting this, the GAG-rich DMECM-I markedly reduced endothelial activation (Figure 9), aligning with evidence that high GAG density limits endothelial migration and sprouting [19]. Inner meniscus ECM also suppresses endothelial PPAR signaling [22], providing a meniscus-specific inhibitory mechanism.

In contrast, the outer meniscus contains COL1- and fibronectin-rich fibrillar networks that provide tensile stiffness and integrin-binding sites, supporting endothelial activation and fibrovascular remodeling. Accordingly, DMECM-O permitted sprouting in Figure 9, and LOX enrichment may further enhance collagen crosslinking and matrix stiffness [22].

The middle region functions as a biochemical and mechanical transition zone. Native red–white regions exhibit partial vascularity and bridge the fibrovascular periphery with the avascular core [4]. DMECM-M reflected this state, exhibiting intermediate GAG content and high abundance of SLRPs (FMOD, DCN, CHAD, BGN, PRELP) as well as THBS1, consistent with its transitional biochemical profile [6]. These proteins regulate collagen fibrillogenesis and restrain excessive angiogenesis in fibrocartilaginous matrices [20, 21], likely moderating—rather than fully inhibiting—endothelial entry, producing the intermediate response observed in Figure 9.

Collectively, DMECM-I/M/O establish an “inhibitory–regulatory–permissive” angiogenic gradient paralleling the native meniscus. DMECM-I forms a GAG-rich anti-angiogenic niche, DMECM-M provides a transitional matrix that tempers vascular infiltration and DMECM-O supports a fibrovascular-permissive environment. This graded angiogenic regulation, together with the zone-specific fibrochondrogenic cues demonstrated earlier, offers a coherent mechanistic basis for the spatially patterned regeneration achieved by the DBM + DMECM-I/M/O scaffold. The subcutaneous model was intentionally selected to analyze such angiogenic function, as it provided a controlled environment to isolate and evaluate the scaffold’s intrinsic angiogenic and anti-angiogenic cues without the confounding mechanical and inflammatory inputs present in the joint.

### Structural and biological significance of the DBM scaffold

The DBM scaffold served as the structural backbone of the composite construct, providing mechanical robustness, reproducibility and compatibility with hydrogel compartmentalization. DBM retains its native collagenous microarchitecture and porosity, supporting cell infiltration and remodeling without the need for additional biochemical modifiers or synthetic crosslinkers [11]. As shown in Figure 1A–H, the decellularization and decalcification processes preserved the trabecular-like porosity, pore size distribution and overall ultrastructural morphology, while maintaining low residual DNA and retaining a basal level of collagen. The compressive modulus of DBM (Figure 1I) further confirmed that the scaffold provides sufficient stiffness to function as a load-bearing framework. In addition, the preserved collagen network may transmit microscale mechanical cues to resident cells, guiding matrix alignment and integrative remodeling across zones.

Unlike synthetic polymer scaffolds that require chemical modification, DBM maintains its intrinsic bioactivity, mechanical integrity and low immunogenicity [10, 11]. Standardized tissue-processing workflows support manufacturing consistency and scalability. Most importantly, the long-standing clinical use of DBM provides a safety profile that enhances its translational potential relative to many other biomaterials. Thus, DBM functions not merely as a physical framework but as a biologically compatible platform that enables stable hydrogel loading and zone-specific remodeling. Its synergy with DMECM hydrogels integrates structural stability with region-specific biochemical complexity, thereby strengthening the translational relevance of the DBM + DMECM composite scaffold.

### Composite scaffold design and biofabrication strategy

A variety of engineering strategies have been explored to reproduce meniscal zonation. For example, 3D printing offers geometric precision [23], electrospun scaffolds recapitulate fibrous architectures [24] and multilayer or gradient hydrogels attempt to mimic biochemical transitions [9]. However, most approaches still struggle to simultaneously achieve load-bearing mechanical strength, physiologic porosity and region-specific biochemical fidelity—key requirements for functional fibrocartilage regeneration. These limitations highlight the need for a biomaterial system that is mechanically competent, biologically instructive and manufacturable using clinically compatible processes.

To address these limitations, we developed a triphasic composite integrating a preserved DBM framework with region-specific DMECM hydrogels. The DBM was shaped into an anatomically relevant triangular prism (Figure 4J), providing defined spatial boundaries for zonal hydrogel localization. Optimization studies demonstrated that a 2% DMECM formulation provided optimal viscosity, photoreactivity and ECM density for controlled DBM infiltration (Figure 3A–D). Five sequential coating cycles generated a plateau in mass gain while maintaining porosity and compressive modulus (Figure 4B–G), yielding reproducible zone-dependent collagen and GAG distributions within the composite scaffold (Figure 4H and I).

The manufactured constructs retained geometric stability after implantation (Figure 6A) and supported early functional remodeling, as evidenced by increased collagen/GAG deposition and improved compressive modulus (Figure 6B–D). These findings suggest that the biofabrication sequence generates constructs capable of sustaining early mechanical demands while promoting matrix biosynthesis, features relevant not only for focal repair but also for larger segmental or near-total meniscus reconstruction.

Overall, the integration of DBM geometry, optimized DMECM formulation and standardized sequential coating establishes a reproducible and clinically compatible biofabrication platform. These quantifiable manufacturing parameters—crosslinking kinetics, hydrogel loading efficiency and mechanical outputs—may serve as critical quality attributes for future scale-up and clinical translation.

### Clinical and translational potential

The DBM + DMECM scaffold integrates components that are already familiar within clinical and regulatory frameworks. DBM has established use in orthopedic and spine applications [10, 11] and riboflavin-based photo-crosslinking has been clinically utilized in ophthalmology [25, 26] and extensively studied in dentistry [27]. Region-specific DMECM, derived from decellularized porcine meniscus, can be integrated into established tissue-processing pipelines, supporting standardized and batch-controlled manufacturing [28]. Collectively, these features provide a practical translational foundation built on materials and workflows with established precedents in human use.

The modular triphasic architecture provides broad surgical adaptability. Each DMECM compartment is spatially confined within the DBM framework, allowing defect-specific reconstruction—from focal lesions to extensive or near-total defects—while maintaining the internal zonation necessary for biomechanical and biological function. Given that APM creates a nonregenerating tissue void, a scaffold that reestablishes zonal matrix and vascular cues is particularly well suited for reconstructing partial meniscectomy defects. The scaffold is compatible with standard fixation approaches, including sutures, anchors and adjuvant biologics such as PRP- or MSC-based therapies and the DBM base offers immediate load-bearing capacity. Importantly, the outer zone’s pro-angiogenic potential may improve graft–host integration compared with current meniscal implants. This may help address the reported failure rates of commercially available synthetic meniscal implants, which range from 11.8% to 18% [29].

Together, these attributes position the DBM + DMECM scaffold as a clinically viable, defect-tailorable and potentially off-the-shelf platform that integrates scalable manufacturing with biologically meaningful zonation to enable functional meniscal reconstruction.

### Limitations and future directions

Despite promising outcomes, several aspects require further investigation. Future work should evaluate long-term degradation, immune responses and mechanical durability under physiologic loading, ideally in orthotopic large-animal models. Further refinement of microscale zonal interfaces through advanced biofabrication approaches, together with single-cell and spatial omics analyses, may help clarify DBM + DMECM interactions during remodeling. Integration with patient-specific imaging could support customized scaffold fabrication, while systematic immunological and safety assessments of porcine-derived DMECM will be essential for regulatory progression. Addressing these factors will deepen mechanistic understanding and facilitate translation of the DBM + DMECM scaffold toward patient-specific meniscal repair.

In addition, the current fabrication process involves sequential hydrogel coating steps to achieve controlled zonal infiltration of DMECM within the DBM scaffold. Although this strategy enabled precise spatial localization and reproducible hydrogel loading, the multistep process may be labor-intensive and could limit manufacturing scalability. Future engineering approaches, such as automated hydrogel deposition [30] or vacuum-assisted infiltration methods [31], may help streamline fabrication while preserving structural and mechanical properties.

## Conclusion

This study established a triphasic DBM + DMECM scaffold that recapitulates the native structural and biochemical zonation of the meniscus. The scaffold promoted zone-specific MSC differentiation and spatially organized ECM remodeling, resulting in region-dependent fibrocartilaginous matrix formation. In addition, it generated a graded angiogenic response across the inner, middle and outer regions, reproducing the hierarchical organization of native fibrocartilage. Built from clinically proven natural materials, the scaffold offers a translationally relevant platform for patient-specific meniscus repair and broader gradient-based tissue regeneration.

## Supplementary Material

rbag081_Supplementary_Data
