# Supplementary material for: Zonally patterned demineralized bone matrix–meniscus ECM composite scaffold directing region-specific fibrochondrogenic and angiogenic responses
Source: Regen Biomater. 2026 Apr 26;13:rbag081. doi: 10.1093/rb/rbag081 (PMC13200060; doi:10.1093/rb/rbag081)
Supplement: rbag081_Supplementary_Data [file rbag081_supplementary_data.docx]

**Supplementary data**

**Zonally patterned demineralized bone matrix–meniscus ECM composite scaffold directing region-specific fibrochondrogenic and angiogenic responses**

Hee-Woong Yun^1,2^, Chae-Won Yun^2,3^, Mi Jeong Kim^2,3^, Yeeun Kim^2,3^, Gaeun Shim^2,3^, Sujin Noh^2,4^,

Ho Jin Lee^2^, Sumin Lim^1^, Jun Young Chung^1^, Jae-Young Park^5^, Do Young Park^1,2,3*^

^1^Department of Orthopedic Surgery, School of Medicine, Ajou University, Suwon, Korea

^2^Cell Therapy Center, Ajou University Medical Center, Suwon, Korea

^3^Ajou University, Leading Convergence of Healthcare and Medicine, Institute of Science & Technology (ALCHeMIST), Suwon, Korea

^4^Department of Biomedical Sciences, Graduate School, Ajou University, Suwon-si, Republic of Korea

^5^Department of Orthopaedic Surgery, CHA University, CHA Bundang Medical Center, Seongnam-si, Korea

* Corresponding author at: Department of Orthopedic Surgery, School of Medicine, Ajou University, 164 World Cup-ro, Yeongtong-gu, Suwon 16499, Republic of Korea.

*E-mail address:* theboy@ajou.ac.kr (D.Y. Park).

Hee-Woong Yun and Chae-Won Yun are equal first authors.

**Supplementary Table S1. Primer sequences used for RT-qPCR analysis.**

| **Primers** | **Sequences** | **Length**  **(bp)** | **Annealing Temp.**  **(℃)** |
| --- | --- | --- | --- |
| *GAPDH* | F: 5’-TGCACCACCAACTGCTTAGC-3’  R: 5’-GGCATGGACTGTGGTCATGAG-3’ | 87 | 60 |
| *COL1A1* | F: 5’-AATGGAGATGATGGGGAAG-3’ R: 5’-CAAACCACTGAAACCTCTG-3’ | 135 | 60 |
| *TNC* | F: 5’- ATGTCCTCCTGACAGCCGAGAA-3’ R: 5’- AGTCACGGTGAGGTTTTCCAGC-3’ | 101 | 60 |
| *COL2A1* | F: 5’-CTCCTGGAGCATCTGGAGAC-3’ R: 5’-ACCACGATCACCCTTGACTC-3’ | 152 | 60 |
| *ACAN* | F: 5’-CCCCACTGGCCCCAAGAATCAAG-3’ R: 5’-CGCTGCGCCCTGTCAAAGTCG-3’ | 319 | 60 |
| *SOX9* | F: 5’-CACACAGCTCACTCGACCTTG-3’ R: 5’-TTCGGTTATTTTTAGGATCATCTCG-3’ | 76 | 60 |

**
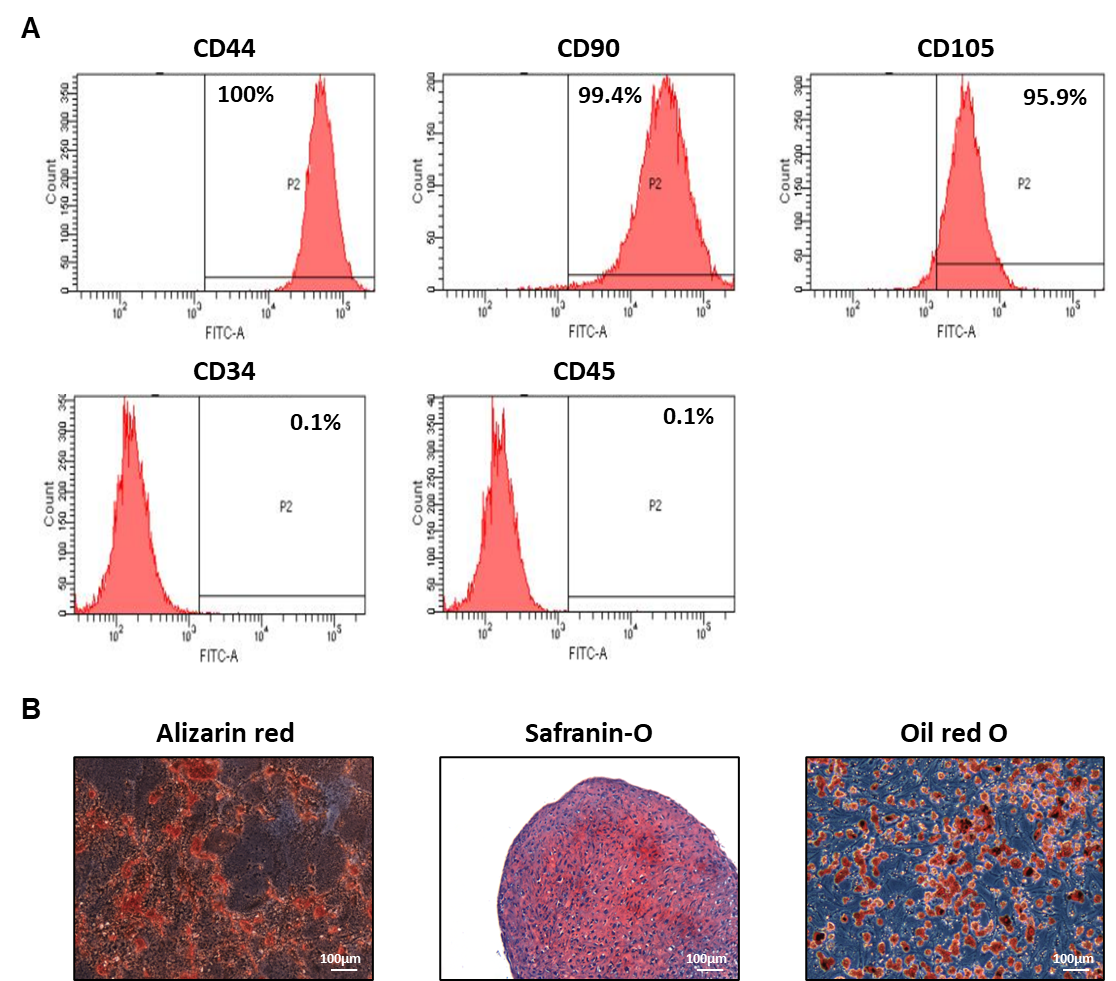
**

**Supplementary Figure S1. Characterization of human synovial membrane–derived mesenchymal stem cells (MSCs).** (A) Immunophenotypic analysis of MSCs showing positivity for CD44, CD90, and CD105 and negativity for CD34 and CD45. (B) Trilineage differentiation capacity of MSCs.

**
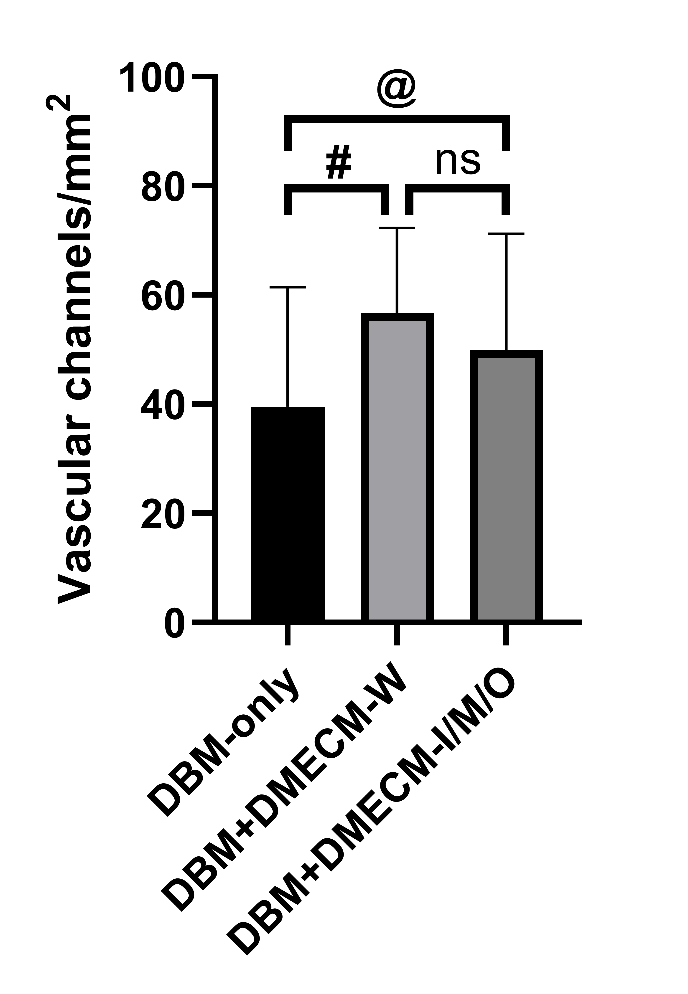
**

**Supplementary Figure S2. Quantitative comparison of total vessel density among groups.** Vessel counts from all regions (inner, middle, and outer) were integrated for each scaffold to evaluate overall angiogenic responses. Data are expressed as mean ± SD (n = 3). Statistical significance was evaluated using one-way ANOVA with Tukey’s multiple comparisons test (*^@^p* < 0.01, *^#^p* < 0.001).

**
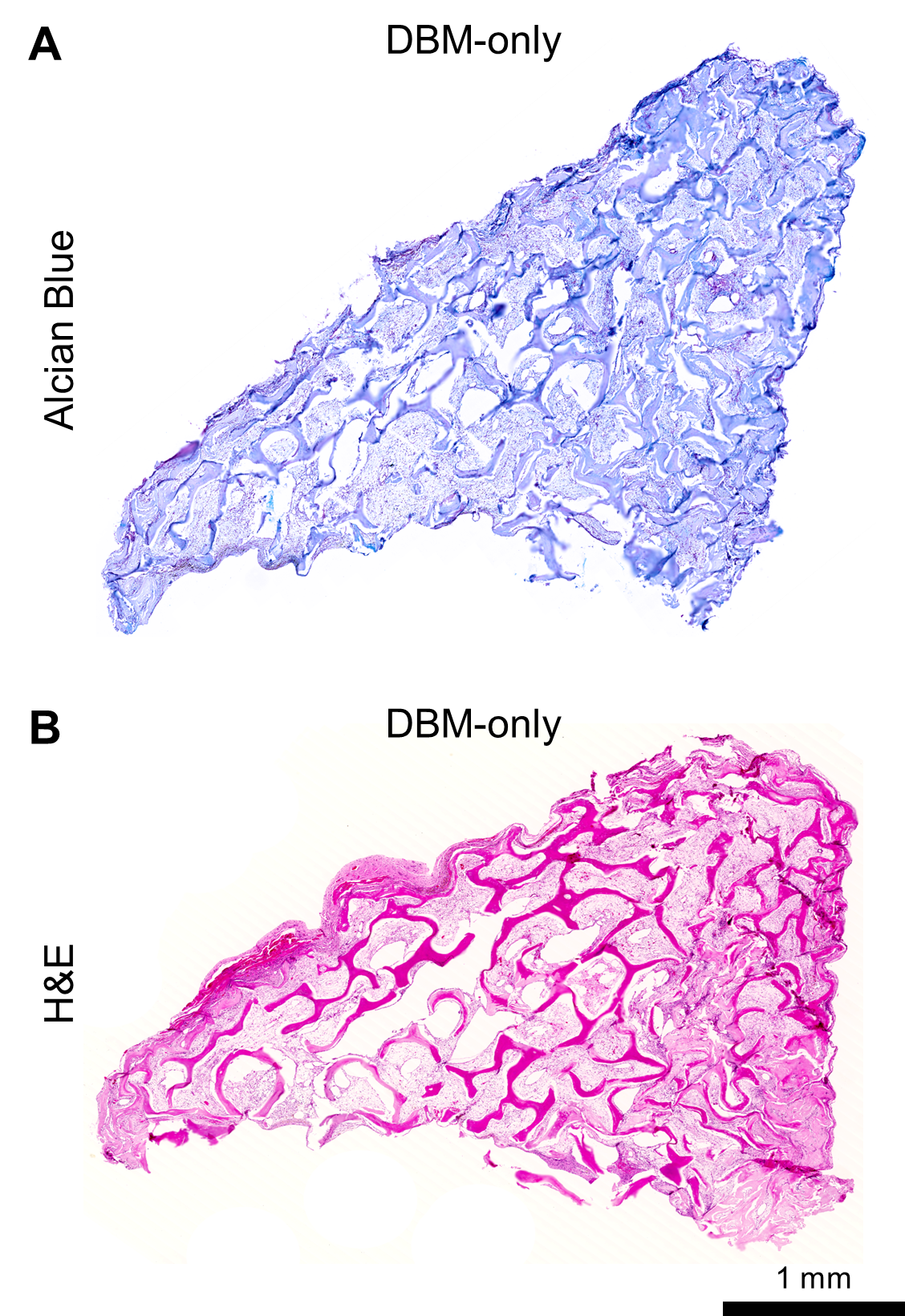
**

**
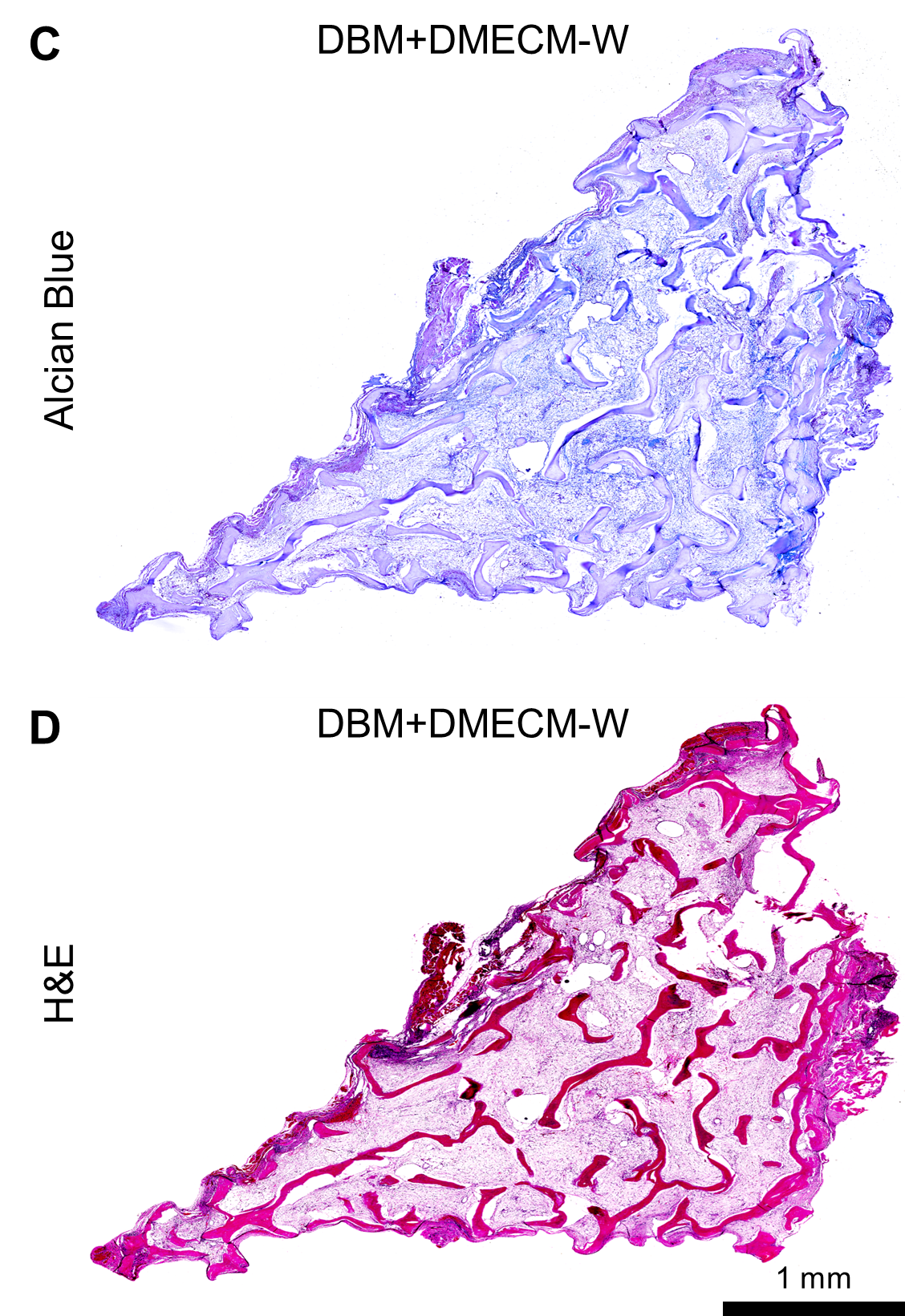
**

**
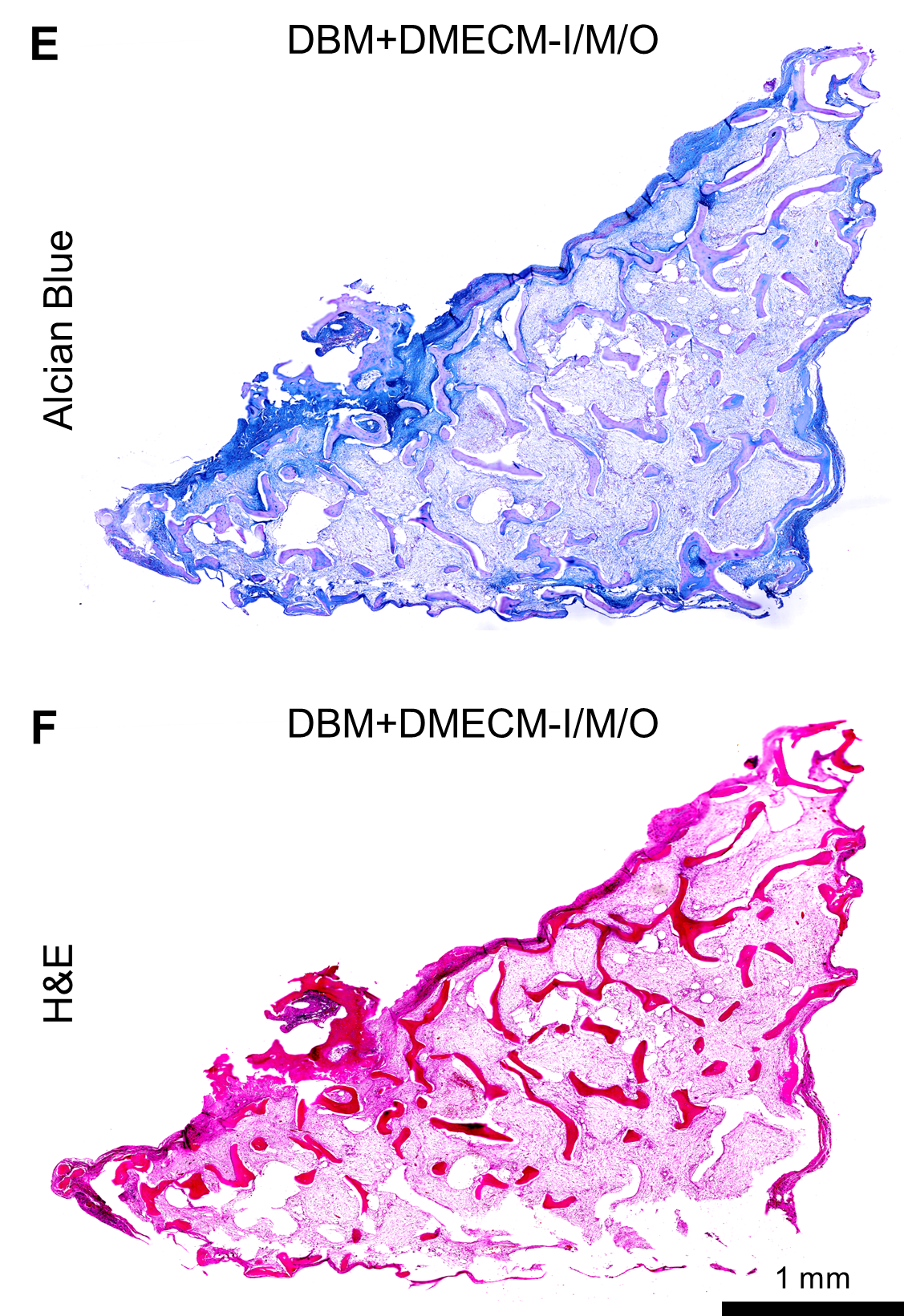
**

**Supplementary Figure S3. Representative panoramic images of explanted scaffolds.** Whole-slide images stained with Alcian Blue and H&E are shown for each experimental group to visualize the spatial distribution of fibrocartilaginous matrix deposition and vascular infiltration within the scaffolds. (A, B) DBM; (C, D) DBM + DMECM-W; (E, F) DBM + DMECM-I/M/O. Alcian Blue staining is shown in A, C, and E, and the corresponding H&E-stained sections are shown in B, D, and F. Scale bars = 1 mm.
